# Comparison of the Impact between Classical and Novel Strains of Rabbit Haemorrhagic Disease on Wild Rabbit Populations in Spain

**DOI:** 10.3390/biology12050728

**Published:** 2023-05-16

**Authors:** Simone Santoro, Juan Antonio Aguayo-Adán, Carlos Rouco

**Affiliations:** 1 Departamento de Ciencias Integradas, Facultad de Ciencias Experimentales, Universidad de Huelva, 21071 Huelva, Spain; simonesantoro77@gmail.com; 2 Departamento de Botánica, Ecología y Fisiología Vegetal, Área de Ecología, Universidad de Córdoba, 14006 Códoba, Spain; jaguayoadan@gmail.com; 3 Departamento Biología Vegetal y Ecología, Área de Ecología, Universidad de Sevilla, 41012 Sevilla, Spain

**Keywords:** lagovirus, GI.1, GI.2, *Oryctolagus cuniculus*, RHDV, RHDV2, RHDVb

## Abstract

**Simple Summary:**

The emergence of two strains of rabbit haemorrhagic disease (i.e., GI.1 and GI.2) in the1990s and 2010s, respectively, has been the primary determinant for the decline of wild European rabbits within their native range. We compared the impact of both strains on the wild rabbit populations in Spain using national hunting bags. Our findings showed that GI.1 had a greater impact on wild rabbit populations than GI.2. This disparity is likely to be explained by several factors, such as climatic conditions, host resistance improvement, virulence attenuation, and population density, among others.

**Abstract:**

The outbreaks of two strains of rabbit haemorrhagic disease (RHD) (GI.1 and GI.2) in the Iberian Peninsula have caused substantial economic losses in commercial rabbitries and have affected the conservation of rabbit-sensitive predators due to the dramatic decline of their natural populations. However, the assessment of the impact of both RHD strains on wild rabbit populations has been limited to a few small-scale studies. Little is known about the overall impact within its native range. In this study, we described and compared the effects of GI.1 and GI.2 countrywide by using time series of hunting bag data widely available across the country and compared their trend during the first eight years after the first outbreak of GI.1 (i.e., 1998) and GI.2 (i.e., 2011), respectively. We used Gaussian generalised additive models (GAM) with the number of hunted rabbits as the response variable and year as the predictor to evaluate the non-linear temporal dynamics of the population at the national and regional community levels. The first GI.1 caused a population decline of around 53%, affecting most Spanish regional communities where the disease occurred. The positive trend observed after GI.1 in Spain ended with the initial outbreak of GI.2, which did not appear to cause a national population decline. In contrast, we found significant variability in the rabbit population trend among regional communities, where some increased, and others decreased. Such a disparity is unlikely to be explained by a single factor; rather, it appears to result from several factors, such as climatic conditions, host resistance improvement, virulence attenuation, or population density. Our study suggests that a national comprehensive hunting bag series could aid in elucidating the differences in the impact of emerging diseases on a large scale. Future research should focus on national longitudinal serological studies to shed light on the immunological status of rabbit populations in different regions to better understand the evolution of RHD strains and the resistance gained by the wild populations.

## 1. Introduction

The European wild rabbit (*Oryctolagus cuniculus*, henceforth “wild rabbit”) is a multifunctional keystone species in Iberian Mediterranean ecosystems [1]. It is the main prey for more than 30 Iberian predators, including highly threatened top predators such as the Iberian lynx (*Lynx pardinus*) and the Spanish Imperial eagle (*Aquila adalberti*), within their native range (i.e., the Iberian Peninsula) [1]. In addition, wild rabbit is historically the most important hunting species in Spain, both in the number of animals hunted (>5.5 million yearly) and in the economic value generated [2]. However, wild rabbit populations have declined during the last 70 years, causing a negative impact on the conservation of threatened species that depend on rabbits, as well as on the hunting-based economy [3]. Among the causes for such a decline, predation, hunting, habitat loss, and diseases are the most important. The emergence of two viral diseases, myxomatosis in the 1950s and two different strains of rabbit haemorrhagic disease (RHD) at the end of the 1980s and 2010 [3,4,5], respectively, have reduced rabbit populations in Spain and Portugal to levels that are affecting ecosystem functioning [6,7]. As a result, the European wild rabbit is considered endangered within its native range [8].

Rabbit haemorrhagic disease is an acute and usually fatal, necrotising form of viral hepatitis that affects wild and domestic rabbits. RHD is caused by a virus belonging to the Caliciviridae family (*Lagovirus europaeus*/*GI.1*/, according to the nomenclature proposed by [9]; henceforth GI.1), which was first detected in China in 1984 [10]. Following its appearance in Europe in 1986, it spread quickly through the Iberian Peninsula. The first outbreaks occurred in 1988 in Spain and 1989 in Portugal (reviewed in [11,12]). Wherever this disease appeared, it caused a significant decrease in rabbit numbers (e.g., case fatality rates in adults frequently exceeded 90%) and, in some cases, the extinction of local rabbit populations (reviewed in [11]). Later, RHD became enzootic, and mortality rates decreased, although continuing to play an essential role in shaping the dynamics of the wild rabbit populations [13]. Until 2010, all RHDV strains circulating in natural European rabbit populations belonged to the same genogroup (G1). However, in 2010, a novel lagovirus affecting rabbits was identified in France [14]. This new lagovirus showed a distinct antigenic profile [14,15]. This new strain of RHDV was initially described as RHDV2 [15] or RHDVb [16]. However, following the nomenclature based on phylogenetic relationships, this new strain was designated as *L. europaeus*/*GI.2*, henceforth GI.2 [9]. GI.2 reached Spain in 2011 [16] and spread rapidly across Europe and beyond in only a few years [17], slightly faster than GI.1 [12].

The GI.1 and new GI.2 lagoviruses share some epidemiological characteristics but also have striking differences. GI.2 has a lower case fatality rate than GI.1 (40% vs. 90%) [11,14,15]. One of the most critical differences regards the susceptibility across age classes. Although an increase in the pathogenicity of the new lagovirus in adult rabbits has been detected [18], GI.2 appears to be less virulent for adult rabbits (0–75% mortality [15]) when compared to mortality caused by the first GI.1 outbreak (above 90% [11]). GI.2 affects rabbits of different age classes, including kittens of just 11 days [12] and several European hare species such as *Lepus granatensis*, *Lepus europaeus*, *Lepus capensis* and *Lepus timidus* [19,20,21,22]. More recently, GI.2 has also been detected in wild black-tailed jackrabbits (*Lepus californicus*) and wild desert cottontail rabbits (*Sylvilagus audubonii*) in North America [5] and even in badgers (*Meles meles*) in Portugal [23]. Furthermore, GI.2 acquired an incredible diversity by recombining with pathogenic and non-pathogenic forms, and currently, several GI.2 recombinants are co-circulating in the wild [24], possibly replacing the former GI.1 strain [4,25].

The outbreak of GI.2 in the Iberian Peninsula has caused substantial economic losses in rabbitries [26] and has affected the conservation of rabbit-sensitive predators due to the dramatic decline of their natural populations [6,7]. However, the assessment of the impact of both RHD strains on wild rabbit populations within its native range has been narrowed to a few studies at a local scale (e.g., [7,27,28,29,30,31,32,33]), and little is known about the overall impact that both RHD strains original outbreaks have had on the wild populations at a larger scale. To enhance our understanding of RHD outbreaks, we need long-term and empirical studies to account for abundance fluctuations and understand the disease’s demographic impact. This is important because it can provide an overall view of the real impact that both strains have had on the Iberian rabbit populations as a whole. This knowledge may assist future studies in predicting the trend that wild populations may have in the future. Thus, in this study, we aim to describe and compare the impact of GI.1 and GI.2 countrywide. Long-term, accurate and cost-effective assessments of population dynamics in this species are challenging [34,35,36]. We used time series of hunting bag data that are widely available across the country and have previously been used to proxy changes in population size at broad time and spatial scales [37]. We compared their trend during the first eight years after the first outbreak of GI.1 and GI.2, respectively.

## 2. Materials and Methods

**Figure 1:**
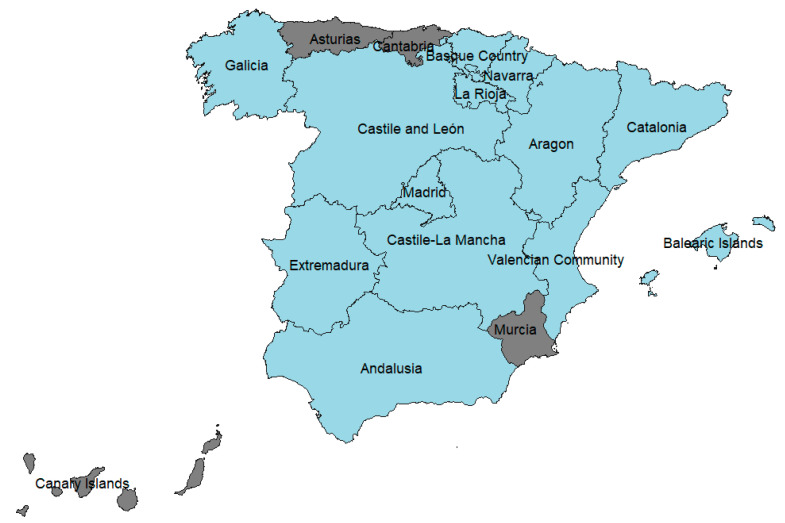
The study area includes the communities with enough hunting bag data to conduct the analyses (in grey, the four communities were excluded from the analyses because of insufficient data).

We collected data on the yearly numbers of hunted rabbits and hunting licenses issued in Spain between 1988 and 2018 (except for 2004, when no data were available) from the Instituto Nacional de Estadística of the Ministerio de Economía, Industria y Competividad (INE) and the web site of the Spanish Ministry of Ecological Transition and Demographic Challenge [2]. Although these data were also available at a national level, we calculated the yearly national values of the number of hunted rabbits (*ynHR*) as the sum of data at the community level (*ycHR*) after excluding four communities with little data available (Asturias and Cantabria with just one and five records over the entire study period, and the Canary Islands and Murcia with zero and five records during the GI.2 outbreak, Figure 1).

Next, we processed these data through two sequential steps to obtain plausible values of *ycHR* when unavailable or implausible according to the overall temporal pattern. First, we used the function *rfImpute* from the *randomForest* package [38] to replace, for each community, the missing observations *ycHR* with the most plausible value according to the relationship with year and the epidemic situation (no RHD/GI.1/GI.2) (for details on proximity analysis, see [39]). Second, we used the *tsoutliers* function, which uses a t-statistic to test the significance of outliers at each time step, from the *forecast* package [40] to identify outliers in the *ycHR* time series and replace them with linearly interpolated values using the neighbouring observations from each regional community.

In a preliminary analysis using the national database, we discarded the hypothesis that the number of hunted rabbits covaried with the number of hunting licenses. One of the most likely explanations for why the number of licenses does not correlate with the number of hunted rabbits is that the same person can have one license per community. Therefore, we interpreted the number of hunted rabbits as a proxy of their population size. We used Gaussian generalised additive models (GAM) with the number of hunted rabbits as the response variable and year as the predictor to evaluate the non-linear temporal dynamics of the population at the national and regional community levels (details in Supplementary Material). A GAM allows the response variable to be modelled as a nonparametric smoothing function of explanatory variables [41], relaxing the generalised linear model’s (GLM) linearity assumption by allowing the data to define the shape of the relationship. We used a GAM approach because population dynamics over time are typically non-linear, particularly when epidemic outbreaks (in this study, GI.1 and GI.2) affect animal abundance [42].

We used Gaussian GAMs and GLMs to assess the temporal variation of *ynHR* at the national and community levels and evaluate the impact of GI.1 and GI.2 outbreaks. We took a two-step approach. The first step was to use GAMs to assess the non-linear temporal pattern of *ynHR*. All models were fitted using the *mgcv* package [43]. We used the function *gam.check* to select the basic dimension for each predictor based on the estimated degrees of freedom value in the main effect (i.e., *year’s* effect). We tested the effects of GI.1 and GI.2 on *ynHR* in the second step. At the national and community levels, we created a subset for GI.1 and another for GI.2, which included the first eight years after each strain’s outbreak in Spain. We log-transformed data to improve the normality of residuals and limited the data to eight years since the beginning year of each strain’s epidemic because (i) data from such a short period were more likely to meet the linearity assumption and (ii) eight years was the period available (at the time we ran the analyses) since the start of GI.2, allowing us to compare their effects. Then, we fitted a GLM to the subset datasets at the national and community levels to test whether *ynHR* decreased after the strain appeared. Notably, for the years 1988–1995 and 2011–2018, when we evaluated the effects of GI.1 and GI.2, only two of the 208 hunted rabbit values per community and year are missing. We performed all analyses in R version 4.2.1 [44].

## 3. Results

### 3.1. Nationwide Effect

**Figure 2:**
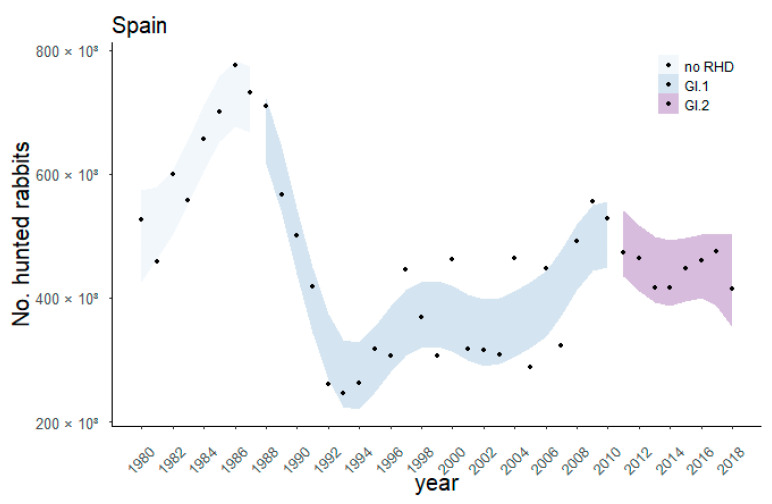
Number of reported hunted rabbits between 1980 and 2018 in Spain. Different colours indicate the 95% CI non-linear trends according to the GAMs fitted to the national data during three periods: 1980–1987 (before GI.2), 1988–2010 (GI.1) and 2011–2018 (GI.2).

During the GI.1 study period (1988–1995), the overall linear trend of the number of hunted rabbits in Spain was significantly negative (*p* < 0.01), whereas no trend was observed during the GI.2 study period (2011–2018, *p* = 0.64; Figure 2).

**Figure 3:**
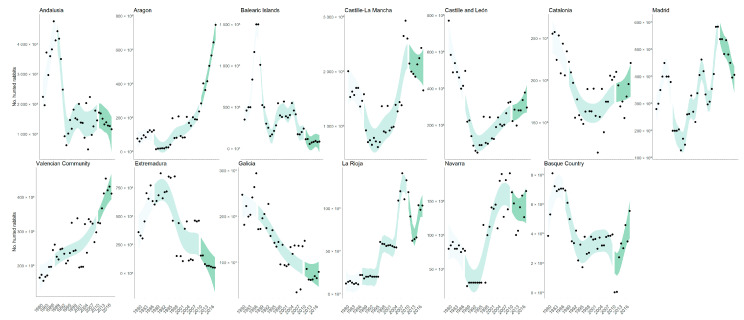
Number of reported hunted rabbits between 1980 and 2018 in thirteen regional communities. Different colours indicate the 95% CI non-linear trends according to the GAMs fitted to the community-level data during three periods: 1980–1987 (before GI.2), 1988–2010 (GI.1) and 2011–2018 (GI.2).

Hunted rabbits declined on average (±95% CI) 53.7% (33.2–74.2%) between 1986 and 1993 after the GI.1 outbreak. In contrast, the decline of hunted rabbits between 2011 and 2018 after the GI.2 outbreak was 0.8%, with a discrepancy among regional communities. In some regions, hunted rabbits declined by more than 30% (lower 95% CI) and in others by more than 29% (upper 95% CI) (Figure 3).

### 3.2. Regionwide Effect

Almost 60% of all the hunted rabbits between 1988 and 2018 were collected in two regional communities: Andalusia (32.7%) and Castille La Mancha (24.8%). We found significant heterogeneity in each community’s GI.1 and GI.2 linear trends (Figure 3, Table 1).

For GI.1, we found a statistically significant decrease in eight out of thirteen communities (Andalusia, Balearic Islands, Castille-La Mancha, Castille and León, Catalonia, Madrid, and Basque Country) with no one showing a linear increase. For GI.2, only two communities showed a significant decrease (Andalusia and Extremadura). In contrast, five others increased significantly (Aragon, Castille and León, Madrid, Valencian Community and Basque Country).

## 4. Discussion

The emergence of myxomatosis and RHD (i.e., GI.1) in the twentieth century, as well as the recent new RHD strain outbreak in 2010 (i.e., GI.2), have been the primary determinants for the decline of wild European rabbits within their native range (e.g., [3,12,45]). According to our findings, rabbit populations in Spain showed a positive trend in the years before the first outbreak of GI.1, when rabbit numbers dropped by around 53%. The observed negative trend at a national level primarily responds to declines in two large regions where nearly 60% of the hunted animals proceed: Andalusia and Castille La Mancha. In contrast, GI.1 significantly impacted most Spanish regional communities where the disease arrived (Table 1). After GI.1, negative trends occurred in all regions except Extremadura. The positive trend we have observed after GI.1 in Spain ended with the initial outbreak of GI.2, which did not appear to cause an overall decline of the population at the national level, as was the case after GI.1. In comparison to what we observed with GI.1, we discovered significant variability in the rabbit population trend among regional communities (Table 1). Populations declined by 30% on average in some regions (e.g., 17% in Castille La Mancha and 31% in Andalusia) while increasing in others (e.g., 80% in Aragon between 2011 and 2018). Similar findings were found in Australia during the GI.1 outbreaks, with more significant rabbit loss in arid areas and less in humid areas [46]. These patterns observed in Australian populations suggest that local differences in rabbit mortality may partly reflect differences in climate. However, we did not find a clear relationship between climatic conditions and rabbit population trends. For example, in traditionally wet areas, such as Galicia, the population size had a negative trend after GI.2, and dryer areas, such as Valencian Community, had a positive trend. Other still unrevealed factors must explain the local variation in the impact of GI.2.

Our findings align with the first outbreaks in Europe that decimated most populations [12], as occurred in other countries such as western Germany and different regions in France [47]. Based on our results, the rabbit population decline in Spain ranged from 33.2 to 74.2% among all the regions, which is consistent with previous studies’ estimates. For example, the first outbreaks killed between 55% and 75% of the European rabbit populations in Doñana National Park [27] and 75% in Navarra (N Spain, [48]) and Alicante (SE Spain, [27]). In Spain, wild rabbit populations decreased by over 50% compared to pre-RHD levels (i.e., before 1998), although with important differences between areas [49]. After the initial impact of GI.1, rabbit populations seemed to be slowly recovering (Figure 2). However, such recovery was uneven. In areas with suitable conditions for rabbits and where conservation measures were adopted to reduce other mortality causes such as predation or hunting, populations recovered faster [3,28,49]. After the first outbreaks, Spain’s overall wild rabbit populations increased slowly. According to a long-term study conducted in Australia, the local wild rabbit population recovery was fueled by increased annual survival from GI.1 infection, which appeared to respond to increased natural resistance against the RHD virus [50]. Others identified the resilience of kittens as a crucial element in explaining rabbit populations to GI.1 [51]. These authors argue that mortality from GI.1 should increase with increasing density due to increased contact between rabbits. However, this would also reduce the median age of infection towards the age at which young rabbits still have maternal antibody protection and, hence, can survive the infection. In that case, the effect of GI.1 on the population size would be reduced because many young rabbits survive and maintain the necessary breeding population. This hypothesis would explain the situation in Spain, where GI.1 severely affected some low-density rabbit populations and not other high-density populations. However, in Europe, wild rabbit populations recovered slower than in Australia after GI.1, and a possible explanation for this difference is that a non-pathogenic calicivirus was present in Australia before GI.1. This calicivirus has not been found in Spain but has been detected in Italy and France [52,53]. This non-lethal calicivirus persisted in most wild rabbits in Australian populations during the GI.1 outbreak [46] and could have provided temporal and partial cross-protection to lethal GI.1 [54].

On the other hand, in Spain, rabbits are both a keystone species for the Mediterranean ecosystem and a pest because of the damage they caused to agricultural production in some regions starting from 2010 onwards [55]. Different rabbit control measures are applied in these regions, and hunters are allowed to kill rabbits all year round [56]. Although the number of rabbits killed during the GI.2 outbreak could reflect a change in hunting policy, it does not seem to explain our results exhaustively. Even though rabbit populations increased in some rabbit-damaged regions, such as Aragon, Castile and Leon, and the Valencian Community, we discovered a negative trend in others with similar hunting policies (e.g., Andalusia and Castilla-La Mancha) [55].

In Andalusia, one of the most relevant Spanish rabbit hunting regions, the first GI.2 case was confirmed in the summer of 2013 and continued with a sharp increase in the number of detected cases during 2014, followed by a decreasing trend in the following years [57]. This uneven temporal distribution could be explained by increased population immunity due to natural immunisation as observed in Portugal for GI.2 [4], and similar to what was suggested for GI.1. Although GI.1 and GI.2 outbreaks were found throughout the year, peak incidence usually occurred during the coldest months [4,27]. Hence, low temperatures seem to relate to the higher incidence of RHD. One possible explanation for the overall reduced incidence of GI.2 in the Spanish rabbit population compared to GI.1 incidence is the rise in the annual minimum temperature (on average 0.16 °C degrees/decade during the last century). Winters after GI.2 were warmer than after GI.1 [58]. However, this alone may hardly explain the remarkable disparity found in GI.2 incidence in the rabbit populations among regions compared to what we observed in GI.1. This discrepancy is more likely related to differences between the two virus strains. Like GI.1, GI.2 also spread rapidly across Europe and beyond in only a few years [4,17], suggesting that transmission routes should be such as those for GI.1. However, GI.1 entered and spread in a completely naive population, whereas GI.2 had to overcome immunity to GI.1 (although it is unlikely to be 100% effective [59]. Additionally, GI.2 has shown extraordinary diversity by recombining with pathogenic and non-pathogenic forms of the RHDVs [25]. This capacity of recombination may have favoured the emergence of recombinant viruses with greater fitness, i.e., broadly a greater reproductive rate (R0). The R0 is defined as the average number of new infections that would arise from a single infectious host introduced into a population of susceptible hosts [60]. Sometimes this is achieved through a decline in virulence, but sometimes it is not. For example, with the introduction of the myxoma virus in Australia, we found an example where the host and parasite drive one another’s evolution [61]. In this case, an initial decline in virulence favoured a greater R0, but further decreases were not. There was a selection in the rabbit–myxomatosis system not for decreased virulence but for increased transmissibility (and hence increased fitness), which happens in this system to be maximised at intermediate grades of virulence [60,62]. Myxoma and RHD viruses had a strong selection for increased transmissibility, but this does not always include viral attenuation (or high virulence). In this sense, evidence for an evolutionary trade-off between virulence and transmissibility has only been seen in the myxoma virus and not for GI.1 [62]. Therefore, if this is the case, the high transmissibility of GI.2 with an attenuation of virulence could be compatible with the results we have obtained.

## 5. Conclusions

Our findings show that GI.1 had a greater impact on wild rabbit populations in Spain than GI.2. Such a disparity is unlikely to be explained by a single factor; rather, it appears to be the result of several factors, including climate, host resistance improvement, virulence attenuation, and population density (pest status). Our study suggests that a national comprehensive hunting bag series could aid in elucidating differences in the impact of viruses on a large scale. Future research should focus on national longitudinal serological studies to shed light on the immunological status of rabbit populations in different regions to better understand the evolution of RHD strains.

## Figures and Tables

**Table 1:** Summary of GLMs results per regional community of the trends of the hunting bags after GI.1 and GI.2 outbreaks, respectively. Note that statistically significant *p*-values are in bold.

		GI.1				GI.2		
Community	Slope	Std. Error	*t*-Value	*p*-Value	Slope	Std. Error	*t*-Value	*p*-Value
Andalusia	−0.25	0.07	−3.38	**0.014**	−0.06	0.01	−8.21	**0.0001**
Aragon	−0.01	0.1	−0.95	0.38	0.13	0.01	10.25	**>0.0001**
Balearic Islands	−0.24	0.04	−5.34	**0.0017**	−0.03	0.04	−0.74	0.489
Valencian Community	−0.01	0.01	−1.68	0.145	0.04	0.01	3.46	**0.0134**
Castille La Mancha	−0.09	0.03	−2.75	**0.033**	−0.003	0.02	−0.19	0.854
Castille and Leon	−0.28	0.06	−4.94	**0.002**	0.06	0.02	2.45	**0.05**
Catalonia	−0.06	0.01	−5.51	**0.002**	0.005	0.02	0.28	0.784
Extremadura	0.03	0.02	1.95	0.098	−0.18	0.03	−5.26	**0.001**
Galicia	−0.003	0.02	−0.16	0.876	−0.06	0.04	−1.52	0.178
La Rioja	−0.001	0.009	−0.12	0.912	0.03	0.04	0.78	0.461
Madrid	−0.283	0.06	−4.93	**0.002**	0.06	0.02	2.44	**0.05**
Navarra	−0.06	0.05	−1.32	0.233	0.01	0.03	0.373	0.722
Basque Country	−0.13	0.03	−3.99	**0.007**	0.72	0.204	3.54	**0.012**

## Data Availability

Data used in this research is freely available from the Spanish Ministry of Ecological Transition and Demographic Challenge website. “Estadística Anual de caza”. https://www.miteco.gob.es/es/biodiversidad/estadisticas/Est_Anual_Caza.aspx (accessed on 20 March 2023).

## Supplementary Materials

The following supporting information can be downloaded at: https://www.mdpi.com/article/10.3390/biology12050728/s1, R Markdown HTML document containing the manuscript’s R code analyses and results.
