# Supplementary material for: Comparison of the Impact between Classical and Novel Strains of Rabbit Haemorrhagic Disease on Wild Rabbit Populations in Spain
Source: Biology (Basel). 2023 May 16;12(5):728. doi: 10.3390/biology12050728 (PMC10215793; doi:10.3390/biology12050728)
Supplement: Supplementary file 1 [file biology-12-00728-s001.zip › biology-2333271-supplementary.html]

Appendix S1


Code 

- Show All Code
- Hide All Code

# Appendix S1

#### 2023-10-28

Loading the required libraries

```
library(mapSpain)
library(randomForest)
library(ggplot2)
library(ggpubr)
library(mgcViz)
library(forecast)
library(DHARMa)
```

We first prepare the database for the communities and, afterwards,
assemble them together to prepare the national database.

```
comunidad<-read.table("comunidad.txt", header = TRUE, sep = "\t",stringsAsFactors = T)
comunidad<- comunidad[names(comunidad[c(1,2,3)])]# eliminating unnecessary columns
```

Eliminating asturias, Cantabria,Asturias and Murcia (because of
insufficient data - see main text)

```
comunidad<- comunidad[comunidad$community!="Asturias"&comunidad$community!="Cantabria"&comunidad$community!="R.deMurcia"&comunidad$community!="Canarias",]
comunidad$community<- factor(comunidad$community)
```

Creating one dataframe for each community

```
for (i in levels(comunidad$community)) {
  command <- paste0(i, "<-subset(comunidad, community=='", i, "')")
  eval(parse(text=command)) 
}
```

For each community, we replace missing values with the estimated ones
from random forest techniques.

This is to identify and replace outliers in the time series of each
community

```
for (i in levels(comunidad$community)) {
  command <- paste0(i,"$N[tsoutliers(",i,"$N)$index]<- tsoutliers(",i,"$N)$replacements") 
  eval(parse(text=command))
}
```

Joining the datasets together again

```
comunidad<- rbind(Andalucia,Aragon,Baleares,C.Valenciana,CastillalaMancha,CastillayLeon,Cataluna,Extremadura,Galicia,LaRioja,Madrid,Navarra,PaisVasco)
comunidad$RHD<- as.factor(ifelse(comunidad$year<1988,"no RHD",ifelse(comunidad$year<2011,"GI.1","GI.2")))
```

Creating a new national dataset made of the sums of annual Ns from
each community (excluding Cantabria, Asturias and Murcia)

```
nacionalnew<- aggregate(comunidad$N, list(comunidad$year), FUN=mean)
names(nacionalnew)<- c("year","N")
```

# National data - GAM analysis

We use GAM analyses on all years of collected data to evaluate the
temporal pattern at the national level in the number of hunted
rabbits.

```
gam1 <- gam(N ~ s(year, k=20) , data=nacionalnew, select=T, method="REML")# select=T means that we are using the double penalty approach to do selection. When select=T the p-value refers to the probability that actually there is no effect at all of the predictor on the response variable (flat line).
```

```
summary(gam1)# summary of results
```

```
## 
## Family: gaussian 
## Link function: identity 
## 
## Formula:
## N ~ s(year, k = 20)
## 
## Parametric coefficients:
##             Estimate Std. Error t value Pr(>|t|)    
## (Intercept)   455500       8649   52.67   <2e-16 ***
## ---
## Signif. codes:  0 '***' 0.001 '**' 0.01 '*' 0.05 '.' 0.1 ' ' 1
## 
## Approximate significance of smooth terms:
##           edf Ref.df     F p-value    
## s(year) 10.21     19 10.94  <2e-16 ***
## ---
## Signif. codes:  0 '***' 0.001 '**' 0.01 '*' 0.05 '.' 0.1 ' ' 1
## 
## R-sq.(adj) =  0.845   Deviance explained = 88.7%
## -REML = 488.63  Scale est. = 2.9173e+09  n = 39
```

```
simgam2<- simulateResiduals(gam1,plot=T)# OK
```

```
gam.check(gam1)# OK
```

```
## 
## Method: REML   Optimizer: outer newton
## full convergence after 6 iterations.
## Gradient range [-3.013763e-06,-6.044938e-07]
## (score 488.6339 & scale 2917332364).
## Hessian positive definite, eigenvalue range [3.000395e-06,20.55868].
## Model rank =  20 / 20 
## 
## Basis dimension (k) checking results. Low p-value (k-index<1) may
## indicate that k is too low, especially if edf is close to k'.
## 
##           k'  edf k-index p-value
## s(year) 19.0 10.2    1.49       1
```

Creating a new, toy dataframe to make predictions from the model and
use them to visualize them. Note there is no data for 2004, because it
was not available (see main text).

```
newdata<- data.frame(year=1980:2018,N=nacionalnew$N)
predizioni<- predict(gam1,newdata = newdata, type="terms",se=T)
newdata$predsgam1<- as.numeric(predizioni$fit)+gam1$coefficients[1]
newdata$predsgam1se<- as.numeric(predizioni$se.fit)
newdata$RHD<- factor(c(rep("no RHD",8),rep("GI.1",23),rep("GI.2",8)))
newdata$RHD<- relevel(newdata$RHD,"no RHD")
```

## National yearly numbers of hunted rabbits

This figure (Figure 2 in main text) represents the GAM curve
depicting the variation in the number of hunted rabbits between 1980 and
2018 before and after GI.1 and GI.2. outbreaks

```
# png("Figure2.png",width=480*1.5)
ggplot(newdata, aes(x = year, y = N, fill=RHD))+
  geom_smooth(aes(ymin=predsgam1-1.96*predsgam1se,ymax=predsgam1+1.96*predsgam1se,fill= RHD),stat="identity",linetype=0,size=5)+
  scale_fill_brewer(palette = "BuPu")+
  geom_point()+
  scale_x_continuous(breaks = seq(min(newdata$year), max(newdata$year), by = 1))+ 
  theme(axis.text.x = element_text(angle = 45,size=13,vjust = 0.2))+#vjust align the labels under the tick (in the center)+
  theme(axis.text.y = element_text(size = rel(1.5), angle = 45, margin = margin(t = 0, r = 10, b = 0, l = 0)))+
  theme(axis.text.x = element_text(size = rel(1.5), angle = 45, margin = margin(t = 5, r = 0, b = 0, l = 0)))+
  labs(x="year", y="# hunted rabbits",title="Spain")+
  theme(axis.title.y = element_text(size = rel(1.8), angle = 90))+ 
  theme(axis.title.x = element_text(size = rel(1.8), angle = 0))+
  theme(plot.title = element_text(size = rel(1.8), angle = 00))+
  theme(legend.position = c(0.9,0.9),legend.text = element_text(size = 12),legend.title=element_blank())+
  theme(plot.margin = margin(t = 1, r = 1, b = 1, l = 1, unit = "cm"))
```

```
# dev.off()
```

# Communities data - GAM analysis

We use GAM analyses on all years of collected data to evaluate the
temporal pattern at level of each community in the number of hunted
rabbits.

## Andalusia

```
Andalucia<- comunidad[comunidad$community=="Andalucia",]

gam1and <- gam(N ~ s(year, k=20) , data=Andalucia, select=T, method="REML")
summary(gam1and)# summary of results
```

```
## 
## Family: gaussian 
## Link function: identity 
## 
## Formula:
## N ~ s(year, k = 20)
## 
## Parametric coefficients:
##             Estimate Std. Error t value Pr(>|t|)    
## (Intercept)  2023011      70096   28.86   <2e-16 ***
## ---
## Signif. codes:  0 '***' 0.001 '**' 0.01 '*' 0.05 '.' 0.1 ' ' 1
## 
## Approximate significance of smooth terms:
##           edf Ref.df     F p-value    
## s(year) 9.451     19 11.69  <2e-16 ***
## ---
## Signif. codes:  0 '***' 0.001 '**' 0.01 '*' 0.05 '.' 0.1 ' ' 1
## 
## R-sq.(adj) =  0.854   Deviance explained =   89%
## -REML = 566.46  Scale est. = 1.9163e+11  n = 39
```

```
simgam2and<- simulateResiduals(gam1and,plot=T)# OK
```

```
gam.check(gam1and)# OK
```

```
## 
## Method: REML   Optimizer: outer newton
## full convergence after 7 iterations.
## Gradient range [-5.364598e-13,2.599589e-06]
## (score 566.4607 & scale 191626541004).
## eigenvalue range [-2.599205e-06,20.323].
## Model rank =  20 / 20 
## 
## Basis dimension (k) checking results. Low p-value (k-index<1) may
## indicate that k is too low, especially if edf is close to k'.
## 
##            k'   edf k-index p-value
## s(year) 19.00  9.45    1.48       1
```

Creating a new, toy dataframe to make predictions from the model and
use them to visualize them.

```
newdataAnd<- data.frame(year=sort(unique(Andalucia$year)),N=Andalucia[order(Andalucia$year),]$N)
predizioniAnd<- predict(gam1and,newdataAnd = newdataAnd, type="terms",se=T)
newdataAnd$predsgam1and<- as.numeric(predizioniAnd$fit)+gam1and$coefficients[1]
newdataAnd$predsgam1andse<- as.numeric(predizioniAnd$se.fit)
newdataAnd$RHD<- Andalucia$RHD
newdataAnd$RHD<- relevel(newdataAnd$RHD,"no RHD")
```

### Andalusia yearly numbers of hunted rabbits

This figure represents the GAM curve depicting the variation in the
number of hunted rabbits between 1980 and 2018 before and after GI.1 and
GI.2 outbreaks in Andalusia.

```
# png("Figure1and.png",width=480*1.5)
p1<- ggplot(newdataAnd, aes(x = year, y = N, fill=RHD))+
  geom_smooth(aes(ymin=predsgam1and-1.96*predsgam1andse,ymax=predsgam1and+1.96*predsgam1andse,fill= RHD),stat="identity",linetype=0,size=5)+
  scale_fill_brewer(palette = "BuGn")+
  geom_point()+
  scale_x_continuous(breaks = seq(min(newdataAnd$year), max(newdataAnd$year), by = 1))+ 
  theme(axis.title.y = element_text(size = rel(1.8), angle = 90))+ 
  theme(axis.title.x = element_text(size = rel(1.8), angle = 0))+
  theme(axis.text.x = element_text(angle = 45,size=13,vjust = 0.2))+#vjust align the labels under the tick (in the center)+
  theme(axis.text.y = element_text(size = rel(1.5), angle = 45, margin = margin(t = 0, r = 10, b = 0, l = 0)))+
  theme(axis.text.x = element_text(size = rel(1.5), angle = 45, margin = margin(t = 5, r = 0, b = 0, l = 0)))+
  labs(y="# hunted rabbits",title="Andalusia")+
  theme(legend.position = c(0.9,0.9),legend.text = element_text(size = 12),legend.title=element_blank())+
  theme(plot.margin = margin(t = 1, r = 1, b = 1, l = 1, unit = "cm"))
p1
```

```
# dev.off()
```

## Aragon

```
Aragon<- comunidad[comunidad$community=="Aragon",]

gam1ara <- gam(N ~ s(year, k=20) , data=Aragon, select=T, method="REML")
summary(gam1ara)# summary of results
```

```
## 
## Family: gaussian 
## Link function: identity 
## 
## Formula:
## N ~ s(year, k = 20)
## 
## Parametric coefficients:
##             Estimate Std. Error t value Pr(>|t|)    
## (Intercept)   182752       5710      32   <2e-16 ***
## ---
## Signif. codes:  0 '***' 0.001 '**' 0.01 '*' 0.05 '.' 0.1 ' ' 1
## 
## Approximate significance of smooth terms:
##           edf Ref.df     F p-value    
## s(year) 7.416     19 50.81  <2e-16 ***
## ---
## Signif. codes:  0 '***' 0.001 '**' 0.01 '*' 0.05 '.' 0.1 ' ' 1
## 
## R-sq.(adj) =  0.962   Deviance explained =   97%
## -REML = 467.58  Scale est. = 1.2718e+09  n = 39
```

```
simgam2and<- simulateResiduals(gam1ara,plot=T)# OK
```

```
gam.check(gam1ara)#OK
```

```
## 
## Method: REML   Optimizer: outer newton
## full convergence after 11 iterations.
## Gradient range [-3.776319e-08,1.247419e-08]
## (score 467.5842 & scale 1271752878).
## Hessian positive definite, eigenvalue range [0.4193064,19.61939].
## Model rank =  20 / 20 
## 
## Basis dimension (k) checking results. Low p-value (k-index<1) may
## indicate that k is too low, especially if edf is close to k'.
## 
##            k'   edf k-index p-value
## s(year) 19.00  7.42    1.16    0.81
```

Creating a new, toy dataframe to make predictions from the model and
use them to visualize them.

```
newdataAra<- data.frame(year=sort(unique(Aragon$year)),N=Aragon[order(Aragon$year),]$N)
predizioniAra<- predict(gam1ara,newdataAra = newdataAra, type="terms",se=T)
newdataAra$predsgam1ara<- as.numeric(predizioniAra$fit)+gam1ara$coefficients[1]
newdataAra$predsgam1arase<- as.numeric(predizioniAra$se.fit)
newdataAra$RHD<- Aragon$RHD
#str(newdataAra$RHD)
newdataAra$RHD<- relevel(newdataAra$RHD,"no RHD")
```

### Aragon yearly numbers of hunted rabbits

This figure represents the GAM curve depicting the variation in the
number of hunted rabbits between 1980 and 2018 before and after GI.1 and
GI.2 outbreaks in Aragon.

```
# png("Figure1ara.png",width=480*1.5)
p2<- ggplot(newdataAra, aes(x = year, y = N, fill=RHD))+
  geom_smooth(aes(ymin=predsgam1ara-1.96*predsgam1arase,ymax=predsgam1ara+1.96*predsgam1arase,fill= RHD),stat="identity",linetype=0)+
  scale_fill_brewer(palette = "BuGn")+
  geom_point()+
  scale_x_continuous(breaks = seq(min(newdataAra$year), max(newdataAra$year), by = 1))+ 
  theme(axis.title.y = element_text(size = rel(1.8), angle = 90))+ 
  theme(axis.title.x = element_text(size = rel(1.8), angle = 0))+
  theme(axis.text.x = element_text(angle = 45,size=13,vjust = 0.2))+#vjust align the labels under the tick (in the center)+
  theme(axis.text.y = element_text(size = rel(1.5), angle = 45, margin = margin(t = 0, r = 10, b = 0, l = 0)))+
  theme(axis.text.x = element_text(size = rel(1.5), angle = 45, margin = margin(t = 5, r = 0, b = 0, l = 0)))+
  labs(y="# hunted rabbits",title="Aragon")+
  theme(legend.position = c(0.9,0.9),legend.text = element_text(size = 12),legend.title=element_blank())+
  theme(plot.margin = margin(t = 1, r = 1, b = 1, l = 1, unit = "cm"))
p2
```

```
# dev.off()
```

## Balearic Islands

```
Baleares<- comunidad[comunidad$community=="Baleares",]

gam1bal <- gam(N ~ s(year, k=20) , data=Baleares, select=T, method="REML")
summary(gam1bal)# summary of results
```

```
## 
## Family: gaussian 
## Link function: identity 
## 
## Formula:
## N ~ s(year, k = 20)
## 
## Parametric coefficients:
##             Estimate Std. Error t value Pr(>|t|)    
## (Intercept)   415756      11530   36.06   <2e-16 ***
## ---
## Signif. codes:  0 '***' 0.001 '**' 0.01 '*' 0.05 '.' 0.1 ' ' 1
## 
## Approximate significance of smooth terms:
##           edf Ref.df     F p-value    
## s(year) 15.53     19 47.34  <2e-16 ***
## ---
## Signif. codes:  0 '***' 0.001 '**' 0.01 '*' 0.05 '.' 0.1 ' ' 1
## 
## R-sq.(adj) =  0.959   Deviance explained = 97.6%
## -REML =  515.4  Scale est. = 5.1843e+09  n = 39
```

```
simgam2and<- simulateResiduals(gam1bal,plot=T)# OK
```

```
gam.check(gam1bal)# OK
```

```
## 
## Method: REML   Optimizer: outer newton
## full convergence after 7 iterations.
## Gradient range [-4.750783e-05,-6.061421e-06]
## (score 515.4009 & scale 5184330900).
## Hessian positive definite, eigenvalue range [4.751164e-05,22.6113].
## Model rank =  20 / 20 
## 
## Basis dimension (k) checking results. Low p-value (k-index<1) may
## indicate that k is too low, especially if edf is close to k'.
## 
##           k'  edf k-index p-value
## s(year) 19.0 15.5    1.42    0.98
```

Creating a new, toy dataframe to make predictions from the model and
use them to visualize them.

```
newdataBal<- data.frame(year=sort(unique(Baleares$year)),N=Baleares[order(Baleares$year),]$N)
predizioniBal<- predict(gam1bal,newdataBal = newdataBal, type="terms",se=T)
newdataBal$predsgam1bal<- as.numeric(predizioniBal$fit)+gam1bal$coefficients[1]
newdataBal$predsgam1balse<- as.numeric(predizioniBal$se.fit)
newdataBal$RHD<- Baleares$RHD
newdataBal$RHD<- relevel(newdataBal$RHD,"no RHD")
```

### Balearic Islands’ yearly numbers of hunted rabbits

This figure represents the GAM curve depicting the variation in the
number of hunted rabbits between 1980 and 2018 before and after GI.1 and
GI.2 outbreaks in Balearic Islands.

```
# png("Figure1bal.png",width=480*1.5)
p3<- ggplot(newdataBal, aes(x = year, y = N, fill=RHD))+
  geom_smooth(aes(ymin=predsgam1bal-1.96*predsgam1balse,ymax=predsgam1bal+1.96*predsgam1balse,fill= RHD),stat="identity",linetype=0)+
  scale_fill_brewer(palette = "BuGn")+
  geom_point()+
  scale_x_continuous(breaks = seq(min(newdataBal$year), max(newdataBal$year), by = 1))+ 
  theme(axis.title.y = element_text(size = rel(1.8), angle = 90))+ 
  theme(axis.title.x = element_text(size = rel(1.8), angle = 0))+
  theme(axis.text.x = element_text(angle = 45,size=13,vjust = 0.2))+#vjust align the labels under the tick (in the center)+
  theme(axis.text.y = element_text(size = rel(1.5), angle = 45, margin = margin(t = 0, r = 10, b = 0, l = 0)))+
  theme(axis.text.x = element_text(size = rel(1.5), angle = 45, margin = margin(t = 5, r = 0, b = 0, l = 0)))+
  labs(y="# hunted rabbits",title="Balearic Islands")+
  theme(legend.position = c(0.9,0.9),legend.text = element_text(size = 12),legend.title=element_blank())+
  theme(plot.margin = margin(t = 1, r = 1, b = 1, l = 1, unit = "cm"))
p3
```

```
# dev.off()
```

## Basque Country

```
PaisVasco<- comunidad[comunidad$community=="PaisVasco",]
gam1pai <- gam(N ~ s(year, k=20) , data=PaisVasco, select=T, method="REML") 
summary(gam1pai)# summary of results
```

```
## 
## Family: gaussian 
## Link function: identity 
## 
## Formula:
## N ~ s(year, k = 20)
## 
## Parametric coefficients:
##             Estimate Std. Error t value Pr(>|t|)    
## (Intercept)   4102.5      142.9   28.71   <2e-16 ***
## ---
## Signif. codes:  0 '***' 0.001 '**' 0.01 '*' 0.05 '.' 0.1 ' ' 1
## 
## Approximate significance of smooth terms:
##           edf Ref.df     F p-value    
## s(year) 8.172     19 6.837  <2e-16 ***
## ---
## Signif. codes:  0 '***' 0.001 '**' 0.01 '*' 0.05 '.' 0.1 ' ' 1
## 
## R-sq.(adj) =  0.774   Deviance explained = 82.2%
## -REML = 328.64  Scale est. = 7.9621e+05  n = 39
```

```
simgam2pai<- simulateResiduals(gam1pai,plot=T)# OK
```

```
gam.check(gam1pai)# OK
```

```
## 
## Method: REML   Optimizer: outer newton
## full convergence after 11 iterations.
## Gradient range [-8.361913e-05,4.423263e-05]
## (score 328.6437 & scale 796211.1).
## Hessian positive definite, eigenvalue range [0.3252916,19.76446].
## Model rank =  20 / 20 
## 
## Basis dimension (k) checking results. Low p-value (k-index<1) may
## indicate that k is too low, especially if edf is close to k'.
## 
##            k'   edf k-index p-value
## s(year) 19.00  8.17    1.07    0.66
```

Creating a new, toy dataframe to make predictions from the model and
use them to visualize them.

```
newdataPai<- data.frame(year=sort(unique(PaisVasco$year)),N=PaisVasco[order(PaisVasco$year),]$N)
predizioniPai<- predict(gam1pai,newdataPai = newdataPai, type="terms",se=T)
newdataPai$predsgam1pai<- as.numeric(predizioniPai$fit)+gam1pai$coefficients[1]
newdataPai$predsgam1paise<- as.numeric(predizioniPai$se.fit)
newdataPai$RHD<- PaisVasco$RHD
newdataPai$RHD<- relevel(newdataPai$RHD,"no RHD")
```

### Basque Country yearly numbers of hunted rabbits

This figure represents the GAM curve depicting the variation in the
number of hunted rabbits between 1980 and 2018 before and after GI.1 and
GI.2 outbreaks in Basque Country.

```
# png("Figure1pai.png",width=480*1.5)
p13<- ggplot(newdataPai, aes(x = year, y = N, fill=RHD))+
  geom_smooth(aes(ymin=predsgam1pai-1.96*predsgam1paise,ymax=predsgam1pai+1.96*predsgam1paise,fill= RHD),stat="identity",linetype=0)+
  scale_fill_brewer(palette = "BuGn")+
  geom_point()+
  scale_x_continuous(breaks = seq(min(newdataPai$year), max(newdataPai$year), by = 1))+ 
  theme(axis.title.y = element_text(size = rel(1.8), angle = 90))+ 
  theme(axis.title.x = element_text(size = rel(1.8), angle = 0))+
  theme(axis.text.x = element_text(angle = 45,size=13,vjust = 0.2))+#vjust align the labels under the tick (in the center)+
  theme(axis.text.y = element_text(size = rel(1.5), angle = 45, margin = margin(t = 0, r = 10, b = 0, l = 0)))+
  theme(axis.text.x = element_text(size = rel(1.5), angle = 45, margin = margin(t = 5, r = 0, b = 0, l = 0)))+
  labs(y="# hunted rabbits",title="Basque Country")+
  theme(legend.position = c(0.9,0.9),legend.text = element_text(size = 12),legend.title=element_blank())+
  theme(plot.margin = margin(t = 1, r = 1, b = 1, l = 1, unit = "cm"))
p13
```

```
# dev.off()
```

## Castille La Mancha

```
CastillalaMancha<- comunidad[comunidad$community=="CastillalaMancha",]
CastillalaMancha<- CastillalaMancha[order(CastillalaMancha$year),]

gam1clm <- gam(N ~ s(year, k=20) , data=CastillalaMancha, select=T, method="REML")
summary(gam1clm)# summary of results
```

```
## 
## Family: gaussian 
## Link function: identity 
## 
## Formula:
## N ~ s(year, k = 20)
## 
## Parametric coefficients:
##             Estimate Std. Error t value Pr(>|t|)    
## (Intercept)  1484833      45925   32.33   <2e-16 ***
## ---
## Signif. codes:  0 '***' 0.001 '**' 0.01 '*' 0.05 '.' 0.1 ' ' 1
## 
## Approximate significance of smooth terms:
##           edf Ref.df     F p-value    
## s(year) 6.636     19 7.187  <2e-16 ***
## ---
## Signif. codes:  0 '***' 0.001 '**' 0.01 '*' 0.05 '.' 0.1 ' ' 1
## 
## R-sq.(adj) =  0.782   Deviance explained =   82%
## -REML =  544.5  Scale est. = 8.2254e+10  n = 39
```

```
simgam2and<- simulateResiduals(gam1clm,plot=T)# OK
```

```
gam.check(gam1clm)# OK
```

```
## 
## Method: REML   Optimizer: outer newton
## full convergence after 4 iterations.
## Gradient range [-1.045278e-05,5.132807e-05]
## (score 544.5039 & scale 82254084987).
## Hessian positive definite, eigenvalue range [8.165634e-06,19.59717].
## Model rank =  20 / 20 
## 
## Basis dimension (k) checking results. Low p-value (k-index<1) may
## indicate that k is too low, especially if edf is close to k'.
## 
##            k'   edf k-index p-value
## s(year) 19.00  6.64    0.85    0.16
```

Creating a new, toy dataframe to make predictions from the model and
use them to visualize them.

```
newdataClm<- data.frame(year=sort(unique(CastillalaMancha$year)),N=CastillalaMancha[order(CastillalaMancha$year),]$N)
predizioniClm<- predict(gam1clm,newdataClm = newdataClm, type="terms",se=T)
newdataClm$predsgam1clm<- as.numeric(predizioniClm$fit)+gam1clm$coefficients[1]
newdataClm$predsgam1clmse<- as.numeric(predizioniClm$se.fit)
newdataClm$RHD<- CastillalaMancha$RHD
newdataClm$RHD<- relevel(newdataClm$RHD,"no RHD")
```

### Castille La Mancha yearly numbers of hunted rabbits

This figure represents the GAM curve depicting the variation in the
number of hunted rabbits between 1980 and 2018 before and after GI.1 and
GI.2 outbreaks in Castille La Mancha.

```
# png("Figure1clm.png",width=480*1.5)
p4<- ggplot(newdataClm, aes(x = year, y = N, fill=RHD))+
  geom_smooth(aes(ymin=predsgam1clm-1.96*predsgam1clmse,ymax=predsgam1clm+1.96*predsgam1clmse,fill= RHD),stat="identity",linetype=0)+
  scale_fill_brewer(palette = "BuGn")+
  geom_point()+
  scale_x_continuous(breaks = seq(min(newdataClm$year), max(newdataClm$year), by = 1))+ 
  theme(axis.title.y = element_text(size = rel(1.8), angle = 90))+ 
  theme(axis.title.x = element_text(size = rel(1.8), angle = 0))+
  theme(axis.text.x = element_text(angle = 45,size=13,vjust = 0.2))+#vjust align the labels under the tick (in the center)+
  theme(axis.text.y = element_text(size = rel(1.5), angle = 45, margin = margin(t = 0, r = 10, b = 0, l = 0)))+
  theme(axis.text.x = element_text(size = rel(1.5), angle = 45, margin = margin(t = 5, r = 0, b = 0, l = 0)))+
  labs(y="# hunted rabbits",title="Castille La Mancha")+
  theme(legend.position = c(0.9,0.9),legend.text = element_text(size = 12),legend.title=element_blank())+
  theme(plot.margin = margin(t = 1, r = 1, b = 1, l = 1, unit = "cm"))
p4
```

```
# dev.off()
```

## Castille and Leon

```
CastillayLeon<- comunidad[comunidad$community=="CastillayLeon",]
CastillayLeon<- CastillayLeon[order(CastillayLeon$year),]
gam1cyl <- gam(N ~ s(year, k=20) , data=CastillayLeon, select=T, method="REML")
summary(gam1cyl)# summary of results
```

```
## 
## Family: gaussian 
## Link function: identity 
## 
## Formula:
## N ~ s(year, k = 20)
## 
## Parametric coefficients:
##             Estimate Std. Error t value Pr(>|t|)    
## (Intercept)   276847      10156   27.26   <2e-16 ***
## ---
## Signif. codes:  0 '***' 0.001 '**' 0.01 '*' 0.05 '.' 0.1 ' ' 1
## 
## Approximate significance of smooth terms:
##           edf Ref.df     F p-value    
## s(year) 5.694     19 11.14  <2e-16 ***
## ---
## Signif. codes:  0 '***' 0.001 '**' 0.01 '*' 0.05 '.' 0.1 ' ' 1
## 
## R-sq.(adj) =  0.848   Deviance explained = 87.1%
## -REML = 485.37  Scale est. = 4.0227e+09  n = 39
```

```
simgam2and<- simulateResiduals(gam1cyl,plot=T)# OK
```

```
gam.check(gam1cyl)# OK
```

```
## 
## Method: REML   Optimizer: outer newton
## full convergence after 9 iterations.
## Gradient range [-6.364965e-06,4.789114e-06]
## (score 485.3667 & scale 4022744860).
## Hessian positive definite, eigenvalue range [0.1925016,19.3626].
## Model rank =  20 / 20 
## 
## Basis dimension (k) checking results. Low p-value (k-index<1) may
## indicate that k is too low, especially if edf is close to k'.
## 
##            k'   edf k-index p-value
## s(year) 19.00  5.69    1.02    0.44
```

Creating a new, toy dataframe to make predictions from the model and
use them to visualize them.

```
newdataCyl<- data.frame(year=sort(unique(CastillayLeon$year)),N=CastillayLeon[order(CastillayLeon$year),]$N)
predizioniCyl<- predict(gam1cyl,newdataCyl = newdataCyl, type="terms",se=T)
newdataCyl$predsgam1cyl<- as.numeric(predizioniCyl$fit)+gam1cyl$coefficients[1]
newdataCyl$predsgam1cylse<- as.numeric(predizioniCyl$se.fit)
newdataCyl$RHD<- CastillayLeon$RHD
newdataCyl$RHD<- relevel(newdataCyl$RHD,"no RHD")
```

### Castille and Leon yearly numbers of hunted rabbits

This figure represents the GAM curve depicting the variation in the
number of hunted rabbits between 1980 and 2018 before and after GI.1 and
GI.2 outbreaks in Castille and Leon.

```
# png("Figure1cyl.png",width=480*1.5)
p5<- ggplot(newdataCyl, aes(x = year, y = N, fill=RHD))+
  geom_smooth(aes(ymin=predsgam1cyl-1.96*predsgam1cylse,ymax=predsgam1cyl+1.96*predsgam1cylse,fill= RHD),stat="identity",linetype=0)+
  scale_fill_brewer(palette = "BuGn")+
  geom_point()+
  scale_x_continuous(breaks = seq(min(newdataCyl$year), max(newdataCyl$year), by = 1))+ 
  theme(axis.title.y = element_text(size = rel(1.8), angle = 90))+ 
  theme(axis.title.x = element_text(size = rel(1.8), angle = 0))+
  theme(axis.text.x = element_text(angle = 45,size=13,vjust = 0.2))+#vjust align the labels under the tick (in the center)+
  theme(axis.text.y = element_text(size = rel(1.5), angle = 45, margin = margin(t = 0, r = 10, b = 0, l = 0)))+
  theme(axis.text.x = element_text(size = rel(1.5), angle = 45, margin = margin(t = 5, r = 0, b = 0, l = 0)))+
  labs(y="# hunted rabbits",title="Castille and Leon")+
  theme(legend.position = c(0.9,0.9),legend.text = element_text(size = 12),legend.title=element_blank())+
  theme(plot.margin = margin(t = 1, r = 1, b = 1, l = 1, unit = "cm"))
p5
```

```
# dev.off()
```

## Catalonia

### Temporal pattern of ynHR in Cataluna.

```
Cataluna<- comunidad[comunidad$community=="Cataluna",]
gam1cat <- gam(N ~ s(year, k=20) , data=Cataluna, select=T, method="REML")
summary(gam1cat)# summary of results
```

```
## 
## Family: gaussian 
## Link function: identity 
## 
## Formula:
## N ~ s(year, k = 20)
## 
## Parametric coefficients:
##             Estimate Std. Error t value Pr(>|t|)    
## (Intercept)   189551       3214   58.98   <2e-16 ***
## ---
## Signif. codes:  0 '***' 0.001 '**' 0.01 '*' 0.05 '.' 0.1 ' ' 1
## 
## Approximate significance of smooth terms:
##           edf Ref.df     F p-value    
## s(year) 3.923     19 3.652  <2e-16 ***
## ---
## Signif. codes:  0 '***' 0.001 '**' 0.01 '*' 0.05 '.' 0.1 ' ' 1
## 
## R-sq.(adj) =  0.646   Deviance explained = 68.3%
## -REML = 438.14  Scale est. = 4.028e+08  n = 39
```

```
simgam2and<- simulateResiduals(gam1cat,plot=T)# OK
```

```
gam.check(gam1cat)# OK
```

```
## 
## Method: REML   Optimizer: outer newton
## full convergence after 4 iterations.
## Gradient range [-0.0001422475,0.0001665832]
## (score 438.1402 & scale 402797146).
## Hessian positive definite, eigenvalue range [0.0001279724,19.21014].
## Model rank =  20 / 20 
## 
## Basis dimension (k) checking results. Low p-value (k-index<1) may
## indicate that k is too low, especially if edf is close to k'.
## 
##            k'   edf k-index p-value
## s(year) 19.00  3.92    0.92    0.22
```

Creating a new, toy dataframe to make predictions from the model and
use them to visualize them.

```
newdataCat<- data.frame(year=sort(unique(Cataluna$year)),N=Cataluna[order(Cataluna$year),]$N)
predizioniCat<- predict(gam1cat,newdataCat = newdataCat, type="terms",se=T)
newdataCat$predsgam1cat<- as.numeric(predizioniCat$fit)+gam1cat$coefficients[1]
newdataCat$predsgam1catse<- as.numeric(predizioniCat$se.fit)
newdataCat$RHD<- Cataluna$RHD
newdataCat$RHD<- relevel(newdataCat$RHD,"no RHD")
```

### Catalonia yearly numbers of hunted rabbits

This figure represents the GAM curve depicting the variation in the
number of hunted rabbits between 1980 and 2018 before and after GI.1 and
GI.2 outbreaks in Catalonia.

```
# png("Figure1cat.png",width=480*1.5)
p6<- ggplot(newdataCat, aes(x = year, y = N, fill=RHD))+
  geom_smooth(aes(ymin=predsgam1cat-1.96*predsgam1catse,ymax=predsgam1cat+1.96*predsgam1catse,fill= RHD),stat="identity",linetype=0)+
  scale_fill_brewer(palette = "BuGn")+
  geom_point()+
  scale_x_continuous(breaks = seq(min(newdataCat$year), max(newdataCat$year), by = 1))+ 
  theme(axis.title.y = element_text(size = rel(1.8), angle = 90))+ 
  theme(axis.title.x = element_text(size = rel(1.8), angle = 0))+
  theme(axis.text.x = element_text(angle = 45,size=13,vjust = 0.2))+#vjust align the labels under the tick (in the center)+
  theme(axis.text.y = element_text(size = rel(1.5), angle = 45, margin = margin(t = 0, r = 10, b = 0, l = 0)))+
  theme(axis.text.x = element_text(size = rel(1.5), angle = 45, margin = margin(t = 5, r = 0, b = 0, l = 0)))+
  labs(y="# hunted rabbits",title="Catalonia")+
  theme(legend.position = c(0.9,0.9),legend.text = element_text(size = 12),legend.title=element_blank())+
  theme(plot.margin = margin(t = 1, r = 1, b = 1, l = 1, unit = "cm"))
p6
```

```
# dev.off()
```

## Extremadura

```
Extremadura<- comunidad[comunidad$community=="Extremadura",]
gam1ext <- gam(N ~ s(year, k=20) , data=Extremadura, select=T, method="REML") 
summary(gam1ext)# summary of results
```

```
## 
## Family: gaussian 
## Link function: identity 
## 
## Formula:
## N ~ s(year, k = 20)
## 
## Parametric coefficients:
##             Estimate Std. Error t value Pr(>|t|)    
## (Intercept)   416371      24435   17.04   <2e-16 ***
## ---
## Signif. codes:  0 '***' 0.001 '**' 0.01 '*' 0.05 '.' 0.1 ' ' 1
## 
## Approximate significance of smooth terms:
##           edf Ref.df     F p-value    
## s(year) 4.496     19 4.595  <2e-16 ***
## ---
## Signif. codes:  0 '***' 0.001 '**' 0.01 '*' 0.05 '.' 0.1 ' ' 1
## 
## R-sq.(adj) =  0.697   Deviance explained = 73.3%
## -REML = 516.32  Scale est. = 2.3286e+10  n = 39
```

```
simgam2and<- simulateResiduals(gam1ext,plot=T)# OK
```

```
gam.check(gam1ext)# OK
```

```
## 
## Method: REML   Optimizer: outer newton
## full convergence after 4 iterations.
## Gradient range [-0.0001309699,8.130931e-05]
## (score 516.3195 & scale 23286293815).
## Hessian positive definite, eigenvalue range [0.0001309406,19.27776].
## Model rank =  20 / 20 
## 
## Basis dimension (k) checking results. Low p-value (k-index<1) may
## indicate that k is too low, especially if edf is close to k'.
## 
##           k'  edf k-index p-value
## s(year) 19.0  4.5    1.06    0.61
```

Creating a new, toy dataframe to make predictions from the model and
use them to visualize them.

```
newdataExt<- data.frame(year=sort(unique(Extremadura$year)),N=Extremadura[order(Extremadura$year),]$N)
predizioniExt<- predict(gam1ext,newdataExt = newdataExt, type="terms",se=T)
newdataExt$predsgam1ext<- as.numeric(predizioniExt$fit)+gam1ext$coefficients[1]
newdataExt$predsgam1extse<- as.numeric(predizioniExt$se.fit)
newdataExt$RHD<- Extremadura$RHD
newdataExt$RHD<- relevel(newdataExt$RHD,"no RHD")
```

### Extremadura yearly numbers of hunted rabbits

This figure represents the GAM curve depicting the variation in the
number of hunted rabbits between 1980 and 2018 before and after GI.1 and
GI.2 outbreaks in Extremadura.

```
# png("Figure1ext.png",width=480*1.5)
p9<- ggplot(newdataExt, aes(x = year, y = N, fill=RHD))+
  geom_smooth(aes(ymin=predsgam1ext-1.96*predsgam1extse,ymax=predsgam1ext+1.96*predsgam1extse,fill= RHD),stat="identity",linetype=0)+
  scale_fill_brewer(palette = "BuGn")+
  geom_point()+
  scale_x_continuous(breaks = seq(min(newdataExt$year), max(newdataExt$year), by = 1))+ 
  theme(axis.title.y = element_text(size = rel(1.8), angle = 90))+ 
  theme(axis.title.x = element_text(size = rel(1.8), angle = 0))+
  theme(axis.text.x = element_text(angle = 45,size=13,vjust = 0.2))+#vjust align the labels under the tick (in the center)+
  theme(axis.text.y = element_text(size = rel(1.5), angle = 45, margin = margin(t = 0, r = 10, b = 0, l = 0)))+
  theme(axis.text.x = element_text(size = rel(1.5), angle = 45, margin = margin(t = 5, r = 0, b = 0, l = 0)))+
  labs(y="# hunted rabbits",title="Extremadura")+
  theme(legend.position = c(0.9,0.9),legend.text = element_text(size = 12),legend.title=element_blank())+
  theme(plot.margin = margin(t = 1, r = 1, b = 1, l = 1, unit = "cm"))
p9
```

## Galicia

```
Galicia<- comunidad[comunidad$community=="Galicia",]

gam1gal <- gam(N ~ s(year, k=20) , data=Galicia, select=T, method="REML") 
summary(gam1gal)# summary of results
```

```
## 
## Family: gaussian 
## Link function: identity 
## 
## Formula:
## N ~ s(year, k = 20)
## 
## Parametric coefficients:
##             Estimate Std. Error t value Pr(>|t|)    
## (Intercept)   144480       5240   27.57   <2e-16 ***
## ---
## Signif. codes:  0 '***' 0.001 '**' 0.01 '*' 0.05 '.' 0.1 ' ' 1
## 
## Approximate significance of smooth terms:
##           edf Ref.df     F p-value    
## s(year) 2.863     19 5.892  <2e-16 ***
## ---
## Signif. codes:  0 '***' 0.001 '**' 0.01 '*' 0.05 '.' 0.1 ' ' 1
## 
## R-sq.(adj) =  0.747   Deviance explained = 76.6%
## -REML = 454.73  Scale est. = 1.0708e+09  n = 39
```

```
simgam2and<- simulateResiduals(gam1gal,plot=T)# OK
```

```
gam.check(gam1gal)# OK
```

```
## 
## Method: REML   Optimizer: outer newton
## full convergence after 4 iterations.
## Gradient range [-0.0003050271,0.0002228541]
## (score 454.7262 & scale 1070779005).
## Hessian positive definite, eigenvalue range [2.951103e-05,19.1133].
## Model rank =  20 / 20 
## 
## Basis dimension (k) checking results. Low p-value (k-index<1) may
## indicate that k is too low, especially if edf is close to k'.
## 
##            k'   edf k-index p-value
## s(year) 19.00  2.86    1.13    0.73
```

Creating a new, toy dataframe to make predictions from the model and
use them to visualize them.

```
newdataGal<- data.frame(year=sort(unique(Galicia$year)),N=Galicia[order(Galicia$year),]$N)
predizioniGal<- predict(gam1gal,newdataGal = newdataGal, type="terms",se=T)
newdataGal$predsgam1gal<- as.numeric(predizioniGal$fit)+gam1gal$coefficients[1]
newdataGal$predsgam1galse<- as.numeric(predizioniGal$se.fit)
newdataGal$RHD<- Galicia$RHD
newdataGal$RHD<- relevel(newdataGal$RHD,"no RHD")
```

### Galicia yearly numbers of hunted rabbits

This figure represents the GAM curve depicting the variation in the
number of hunted rabbits between 1980 and 2018 before and after GI.1 and
GI.2 outbreaks in Galicia.

```
# png("Figure1gal.png",width=480*1.5)
p10<- ggplot(newdataGal, aes(x = year, y = N, fill=RHD))+
  geom_smooth(aes(ymin=predsgam1gal-1.96*predsgam1galse,ymax=predsgam1gal+1.96*predsgam1galse,fill= RHD),stat="identity",linetype=0,size=15)+
  scale_fill_brewer(palette = "BuGn")+
  geom_point()+
  scale_x_continuous(breaks = seq(min(newdataGal$year), max(newdataGal$year), by = 1))+ 
  theme(axis.title.y = element_text(size = rel(1.8), angle = 90))+ 
  theme(axis.title.x = element_text(size = rel(1.8), angle = 0))+
  theme(axis.text.x = element_text(angle = 45,size=13,vjust = 0.2))+#vjust align the labels under the tick (in the center)+
  theme(axis.text.y = element_text(size = rel(1.5), angle = 45, margin = margin(t = 0, r = 10, b = 0, l = 0)))+
  theme(axis.text.x = element_text(size = rel(1.5), angle = 45, margin = margin(t = 5, r = 0, b = 0, l = 0)))+
  labs(y="# hunted rabbits",title="Galicia")+
  theme(legend.position = c(0.9,0.9),legend.text = element_text(size = 12),legend.title=element_blank())+
  theme(plot.margin = margin(t = 1, r = 1, b = 1, l = 1, unit = "cm"))
p10
```

```
# dev.off()
```

## La Rioja

```
LaRioja<- comunidad[comunidad$community=="LaRioja",]

gam1lar <- gam(N ~ s(year, k=20) , data=LaRioja, select=T, method="REML") 
summary(gam1lar)# summary of results
```

```
## 
## Family: gaussian 
## Link function: identity 
## 
## Formula:
## N ~ s(year, k = 20)
## 
## Parametric coefficients:
##             Estimate Std. Error t value Pr(>|t|)    
## (Intercept)    54235       1619   33.51   <2e-16 ***
## ---
## Signif. codes:  0 '***' 0.001 '**' 0.01 '*' 0.05 '.' 0.1 ' ' 1
## 
## Approximate significance of smooth terms:
##           edf Ref.df     F p-value    
## s(year) 12.21     19 28.66  <2e-16 ***
## ---
## Signif. codes:  0 '***' 0.001 '**' 0.01 '*' 0.05 '.' 0.1 ' ' 1
## 
## R-sq.(adj) =  0.935   Deviance explained = 95.6%
## -REML =  429.8  Scale est. = 1.0218e+08  n = 39
```

```
simgam2and<- simulateResiduals(gam1lar,plot=T)# problems
```

```
gam.check(gam1lar)# problems
```

```
## 
## Method: REML   Optimizer: outer newton
## full convergence after 8 iterations.
## Gradient range [-1.147485e-06,-1.032252e-07]
## (score 429.7967 & scale 102180507).
## Hessian positive definite, eigenvalue range [8.29943e-07,21.20956].
## Model rank =  20 / 20 
## 
## Basis dimension (k) checking results. Low p-value (k-index<1) may
## indicate that k is too low, especially if edf is close to k'.
## 
##           k'  edf k-index p-value
## s(year) 19.0 12.2    1.38    0.98
```

Creating a new, toy dataframe to make predictions from the model and
use them to visualize them.

```
newdataLar<- data.frame(year=sort(unique(LaRioja$year)),N=LaRioja[order(LaRioja$year),]$N)# there is no data for 2004! do not care about the warning message!
predizioniLar<- predict(gam1lar,newdataLar = newdataLar, type="terms",se=T)
newdataLar$predsgam1lar<- as.numeric(predizioniLar$fit)+gam1lar$coefficients[1]
newdataLar$predsgam1larse<- as.numeric(predizioniLar$se.fit)
newdataLar$RHD<- LaRioja$RHD
newdataLar$RHD<- relevel(newdataLar$RHD,"no RHD")
```

### La Rioja yearly numbers of hunted rabbits

This figure represents the GAM curve depicting the variation in the
number of hunted rabbits between 1980 and 2018 before and after GI.1 and
GI.2 outbreaks in La Rioja.

```
# png("Figure1lar.png",width=480*1.5)
p11<- ggplot(newdataLar, aes(x = year, y = N, fill=RHD))+
  geom_smooth(aes(ymin=predsgam1lar-1.96*predsgam1larse,ymax=predsgam1lar+1.96*predsgam1larse,fill= RHD),stat="identity",linetype=0)+
  scale_fill_brewer(palette = "BuGn")+
  geom_point()+
  scale_x_continuous(breaks = seq(min(newdataLar$year), max(newdataLar$year), by = 1))+ 
  theme(axis.title.y = element_text(size = rel(1.8), angle = 90))+ 
  theme(axis.title.x = element_text(size = rel(1.8), angle = 0))+
  theme(axis.text.x = element_text(angle = 45,size=13,vjust = 0.2))+#vjust align the labels under the tick (in the center)+
  theme(axis.text.y = element_text(size = rel(1.5), angle = 45, margin = margin(t = 0, r = 10, b = 0, l = 0)))+
  theme(axis.text.x = element_text(size = rel(1.5), angle = 45, margin = margin(t = 5, r = 0, b = 0, l = 0)))+
  labs(y="# hunted rabbits",title="La Rioja")+
  theme(legend.position = c(0.9,0.9),legend.text = element_text(size = 12),legend.title=element_blank())+
  theme(plot.margin = margin(t = 1, r = 1, b = 1, l = 1, unit = "cm"))
p11
```

```
dev.off()
```

```
## null device 
##           1
```

## Madrid

```
Madrid<- comunidad[comunidad$community=="Madrid",]
gam1mad <- gam(N ~ s(year, k=20) , data=Madrid, select=T, method="REML") 
summary(gam1mad)# summary of results
```

```
## 
## Family: gaussian 
## Link function: identity 
## 
## Formula:
## N ~ s(year, k = 20)
## 
## Parametric coefficients:
##             Estimate Std. Error t value Pr(>|t|)    
## (Intercept)   356754       5424   65.77   <2e-16 ***
## ---
## Signif. codes:  0 '***' 0.001 '**' 0.01 '*' 0.05 '.' 0.1 ' ' 1
## 
## Approximate significance of smooth terms:
##           edf Ref.df     F p-value    
## s(year) 13.55     19 23.87  <2e-16 ***
## ---
## Signif. codes:  0 '***' 0.001 '**' 0.01 '*' 0.05 '.' 0.1 ' ' 1
## 
## R-sq.(adj) =  0.923   Deviance explained =   95%
## -REML = 479.51  Scale est. = 1.1474e+09  n = 39
```

```
simgam2and<- simulateResiduals(gam1mad,plot=T)# OK
```

```
gam.check(gam1mad)# OK
```

```
## 
## Method: REML   Optimizer: outer newton
## full convergence after 6 iterations.
## Gradient range [-4.928378e-05,-4.231532e-06]
## (score 479.5116 & scale 1147446061).
## Hessian positive definite, eigenvalue range [4.927972e-05,21.62304].
## Model rank =  20 / 20 
## 
## Basis dimension (k) checking results. Low p-value (k-index<1) may
## indicate that k is too low, especially if edf is close to k'.
## 
##           k'  edf k-index p-value
## s(year) 19.0 13.5    1.35    0.97
```

Creating a new, toy dataframe to make predictions from the model and
use them to visualize them.

```
newdataMad<- data.frame(year=sort(unique(Madrid$year)),N=Madrid[order(Madrid$year),]$N)# there is no data for 2004! do not care about the warning message!
predizioniMad<- predict(gam1mad,newdataMad = newdataMad, type="terms",se=T)
newdataMad$predsgam1mad<- as.numeric(predizioniMad$fit)+gam1mad$coefficients[1]
newdataMad$predsgam1madse<- as.numeric(predizioniMad$se.fit)
newdataMad$RHD<- Madrid$RHD
newdataMad$RHD<- relevel(newdataMad$RHD,"no RHD")
```

### Madrid yearly numbers of hunted rabbits

This figure represents the GAM curve depicting the variation in the
number of hunted rabbits between 1980 and 2018 before and after GI.1 and
GI.2 outbreaks in Madrid.

```
# png("Figure1mad.png",width=480*1.5)
p7<- ggplot(newdataMad, aes(x = year, y = N, fill=RHD))+
  geom_smooth(aes(ymin=predsgam1mad-1.96*predsgam1madse,ymax=predsgam1mad+1.96*predsgam1madse,fill= RHD),stat="identity",linetype=0,size=15)+
  scale_fill_brewer(palette = "BuGn")+
  geom_point()+
  scale_x_continuous(breaks = seq(min(newdataMad$year), max(newdataMad$year), by = 1))+ 
  theme(axis.title.y = element_text(size = rel(1.8), angle = 90))+ 
  theme(axis.title.x = element_text(size = rel(1.8), angle = 0))+
  theme(axis.text.x = element_text(angle = 45,size=13,vjust = 0.2))+#vjust align the labels under the tick (in the center)+
  theme(axis.text.y = element_text(size = rel(1.5), angle = 45, margin = margin(t = 0, r = 10, b = 0, l = 0)))+
  theme(axis.text.x = element_text(size = rel(1.5), angle = 45, margin = margin(t = 5, r = 0, b = 0, l = 0)))+
  labs(y="# hunted rabbits",title="Madrid")+
  theme(legend.position = c(0.9,0.9),legend.text = element_text(size = 12),legend.title=element_blank())+
  theme(plot.margin = margin(t = 1, r = 1, b = 1, l = 1, unit = "cm"))
p7
```

```
# dev.off()
```

## Navarra

```
Navarra<- comunidad[comunidad$community=="Navarra",]
gam1nav <- gam(N ~ s(year, k=20) , data=Navarra, select=T, method="REML") 
summary(gam1nav)# summary of results
```

```
## 
## Family: gaussian 
## Link function: identity 
## 
## Formula:
## N ~ s(year, k = 20)
## 
## Parametric coefficients:
##             Estimate Std. Error t value Pr(>|t|)    
## (Intercept)   101609       3742   27.16   <2e-16 ***
## ---
## Signif. codes:  0 '***' 0.001 '**' 0.01 '*' 0.05 '.' 0.1 ' ' 1
## 
## Approximate significance of smooth terms:
##           edf Ref.df     F p-value    
## s(year) 5.654     19 7.758  <2e-16 ***
## ---
## Signif. codes:  0 '***' 0.001 '**' 0.01 '*' 0.05 '.' 0.1 ' ' 1
## 
## R-sq.(adj) =  0.795   Deviance explained = 82.6%
## -REML = 447.26  Scale est. = 5.4596e+08  n = 39
```

```
simgam2and<- simulateResiduals(gam1nav,plot=T)# OK
```

```
gam.check(gam1nav)# OK
```

```
## 
## Method: REML   Optimizer: outer newton
## full convergence after 5 iterations.
## Gradient range [-5.559075e-06,5.124794e-06]
## (score 447.265 & scale 545964259).
## Hessian positive definite, eigenvalue range [5.558998e-06,19.46269].
## Model rank =  20 / 20 
## 
## Basis dimension (k) checking results. Low p-value (k-index<1) may
## indicate that k is too low, especially if edf is close to k'.
## 
##            k'   edf k-index p-value
## s(year) 19.00  5.65     1.3    0.94
```

Creating a new, toy dataframe to make predictions from the model and
use them to visualize them.

```
newdataNav<- data.frame(year=sort(unique(Navarra$year)),N=Navarra[order(Navarra$year),]$N)
predizioniNav<- predict(gam1nav,newdataNav = newdataNav, type="terms",se=T)
newdataNav$predsgam1nav<- as.numeric(predizioniNav$fit)+gam1nav$coefficients[1]
newdataNav$predsgam1navse<- as.numeric(predizioniNav$se.fit)
newdataNav$RHD<- Navarra$RHD
newdataNav$RHD<- relevel(newdataNav$RHD,"no RHD")
```

### Navarra yearly numbers of hunted rabbits

This figure represents the GAM curve depicting the variation in the
number of hunted rabbits between 1980 and 2018 before and after GI.1 and
GI.2 outbreaks in Navarra.

```
# png("Figure1nav.png",width=480*1.5)
p12<- ggplot(newdataNav, aes(x = year, y = N, fill=RHD))+
  geom_smooth(aes(ymin=predsgam1nav-1.96*predsgam1navse,ymax=predsgam1nav+1.96*predsgam1navse,fill= RHD),stat="identity",linetype=0)+
  scale_fill_brewer(palette = "BuGn")+
  geom_point()+
  scale_x_continuous(breaks = seq(min(newdataNav$year), max(newdataNav$year), by = 1))+ 
  theme(axis.title.y = element_text(size = rel(1.8), angle = 90))+ 
  theme(axis.title.x = element_text(size = rel(1.8), angle = 0))+
  theme(axis.text.x = element_text(angle = 45,size=13,vjust = 0.2))+#vjust align the labels under the tick (in the center)+
  theme(axis.text.y = element_text(size = rel(1.5), angle = 45, margin = margin(t = 0, r = 10, b = 0, l = 0)))+
  theme(axis.text.x = element_text(size = rel(1.5), angle = 45, margin = margin(t = 5, r = 0, b = 0, l = 0)))+
  labs(y="# hunted rabbits",title="Navarra")+
  theme(legend.position = c(0.9,0.9),legend.text = element_text(size = 12),legend.title=element_blank())+
  theme(plot.margin = margin(t = 1, r = 1, b = 1, l = 1, unit = "cm"))
p12
```

```
# dev.off()
```

## Valencian Community

```
Valencia<- comunidad[comunidad$community=="C.Valenciana",]
gam1val <- gam(N ~ s(year, k=20) , data=Valencia, select=T, method="REML") 
summary(gam1val)# summary of results
```

```
## 
## Family: gaussian 
## Link function: identity 
## 
## Formula:
## N ~ s(year, k = 20)
## 
## Parametric coefficients:
##             Estimate Std. Error t value Pr(>|t|)    
## (Intercept)   271192       5956   45.53   <2e-16 ***
## ---
## Signif. codes:  0 '***' 0.001 '**' 0.01 '*' 0.05 '.' 0.1 ' ' 1
## 
## Approximate significance of smooth terms:
##           edf Ref.df    F p-value    
## s(year) 3.368     19 7.75  <2e-16 ***
## ---
## Signif. codes:  0 '***' 0.001 '**' 0.01 '*' 0.05 '.' 0.1 ' ' 1
## 
## R-sq.(adj) =  0.795   Deviance explained = 81.3%
## -REML = 461.12  Scale est. = 1.3836e+09  n = 39
```

```
simgam2and<- simulateResiduals(gam1val,plot=T)# OK
```

```
gam.check(gam1val)# OK
```

```
## 
## Method: REML   Optimizer: outer newton
## full convergence after 9 iterations.
## Gradient range [-1.383943e-06,-1.067039e-07]
## (score 461.118 & scale 1383559590).
## Hessian positive definite, eigenvalue range [0.3645777,19.09443].
## Model rank =  20 / 20 
## 
## Basis dimension (k) checking results. Low p-value (k-index<1) may
## indicate that k is too low, especially if edf is close to k'.
## 
##            k'   edf k-index p-value
## s(year) 19.00  3.37    0.99     0.4
```

Creating a new, toy dataframe to make predictions from the model and
use them to visualize them.

```
newdataVal<- data.frame(year=sort(unique(Valencia$year)),N=Valencia[order(Valencia$year),]$N)
predizioniVal<- predict(gam1val,newdataVal = newdataVal, type="terms",se=T)
newdataVal$predsgam1val<- as.numeric(predizioniVal$fit)+gam1val$coefficients[1]
newdataVal$predsgam1valse<- as.numeric(predizioniVal$se.fit)
newdataVal$RHD<- Valencia$RHD
newdataVal$RHD<- relevel(newdataVal$RHD,"no RHD")
```

### Valencian Community yearly numbers of hunted rabbits

This figure represents the GAM curve depicting the variation in the
number of hunted rabbits between 1980 and 2018 before and after GI.1 and
GI.2 outbreaks in Valencian Community.

```
# png("Figure1val.png",width=480*1.5)
p8<- ggplot(newdataVal, aes(x = year, y = N, fill=RHD))+
  geom_smooth(aes(ymin=predsgam1val-1.96*predsgam1valse,ymax=predsgam1val+1.96*predsgam1valse,fill= RHD),stat="identity",linetype=0)+
  scale_fill_brewer(palette = "BuGn")+
  geom_point()+
  scale_x_continuous(breaks = seq(min(newdataVal$year), max(newdataVal$year), by = 1))+ 
  theme(axis.title.y = element_text(size = rel(1.8), angle = 90))+ 
  theme(axis.title.x = element_text(size = rel(1.8), angle = 0))+
  theme(axis.text.x = element_text(angle = 45,size=13,vjust = 0.2))+#vjust align the labels under the tick (in the center)+
  theme(axis.text.y = element_text(size = rel(1.5), angle = 45, margin = margin(t = 0, r = 10, b = 0, l = 0)))+
  theme(axis.text.x = element_text(size = rel(1.5), angle = 45, margin = margin(t = 5, r = 0, b = 0, l = 0)))+
  labs(y="# hunted rabbits",title="Valencian Community")+
  theme(legend.position = c(0.9,0.9),legend.text = element_text(size = 12),legend.title=element_blank())+
  theme(plot.margin = margin(t = 1, r = 1, b = 1, l = 1, unit = "cm"))
p8
```

```
# dev.off()
```

# National data - GLM analyses of linear trends

As it is often the case for large count data, and according to
preliminary analyses on this dataset, the Gaussian distribution fitted
the data better than a Poisson or Poisson-related one. For this reason
we used GLM with gaussian distribution on log-transformed count (no. of
hunted rabbits) data. We considered the eigth years after the outbreak
of each variant of RHD.

```
RHD1<- nacionalnew[nacionalnew$year>1987&nacionalnew$year<1996,]
RHD2<- nacionalnew[nacionalnew$year>2010,]
RHD1$logN<- log(RHD1$N)
RHD2$logN<- log(RHD2$N)
n1<- lm(logN ~ year, data=RHD1)
summary(n1)
```

```
## 
## Call:
## lm(formula = logN ~ year, data = RHD1)
## 
## Residuals:
##      Min       1Q   Median       3Q      Max 
## -0.30930 -0.06462  0.02676  0.07141  0.31642 
## 
## Coefficients:
##              Estimate Std. Error t value Pr(>|t|)   
## (Intercept) 299.05329   64.36987   4.646  0.00352 **
## year         -0.14371    0.03232  -4.446  0.00435 **
## ---
## Signif. codes:  0 '***' 0.001 '**' 0.01 '*' 0.05 '.' 0.1 ' ' 1
## 
## Residual standard error: 0.2095 on 6 degrees of freedom
## Multiple R-squared:  0.7672, Adjusted R-squared:  0.7284 
## F-statistic: 19.77 on 1 and 6 DF,  p-value: 0.004347
```

We found a significant negative linear trend on the log-scale of the
number of yearly hunted rabbits after the GI.1 outbreak (slope = -0.144,
P = 0.00435).

```
n2<- lm(logN ~ year, data=RHD2)
summary(n2)
```

```
## 
## Call:
## lm(formula = logN ~ year, data = RHD2)
## 
## Residuals:
##      Min       1Q   Median       3Q      Max 
## -0.07410 -0.05740  0.01675  0.04167  0.07662 
## 
## Coefficients:
##              Estimate Std. Error t value Pr(>|t|)
## (Intercept) 22.306794  19.265679   1.158    0.291
## year        -0.004618   0.009563  -0.483    0.646
## 
## Residual standard error: 0.06198 on 6 degrees of freedom
## Multiple R-squared:  0.0374, Adjusted R-squared:  -0.123 
## F-statistic: 0.2331 on 1 and 6 DF,  p-value: 0.6463
```

Vice versa, we found a non-significant linear trend on the log-scale
of the number of yearly hunted rabbits after the GI.2 outbreak (slope =
-0.005, P = 0.64632).

# Communities data - GLM analyses of linear trends

Using the eigth days after the outbreak of each variant of RHD.

## Andalusia

```
RHDand1<- Andalucia[Andalucia$year>1987&Andalucia$year<1996,]
RHDand1<- RHDand1[1:8,]# cutting out the years after the first eight (same number for which we have info on RHDand2)
RHDand2<- Andalucia[Andalucia$year>2010,]
RHDand1$logN<- log(RHDand1$N)
RHDand2$logN<- log(RHDand2$N)
n1and<- lm(logN ~ year, data=RHDand1)
summary(n1and)
```

```
## 
## Call:
## lm(formula = logN ~ year, data = RHDand1)
## 
## Residuals:
##     Min      1Q  Median      3Q     Max 
## -0.7039 -0.1437  0.0925  0.2077  0.6391 
## 
## Coefficients:
##              Estimate Std. Error t value Pr(>|t|)  
## (Intercept) 509.84823  146.39081   3.483   0.0131 *
## year         -0.24876    0.07351  -3.384   0.0148 *
## ---
## Signif. codes:  0 '***' 0.001 '**' 0.01 '*' 0.05 '.' 0.1 ' ' 1
## 
## Residual standard error: 0.4764 on 6 degrees of freedom
## Multiple R-squared:  0.6562, Adjusted R-squared:  0.5989 
## F-statistic: 11.45 on 1 and 6 DF,  p-value: 0.01478
```

We found a significant negative linear trend on the log-scale of the
number of yearly hunted rabbits after the GI.1 outbreak (slope = -0.249,
P = 0.01478).

```
n2and<- lm(logN ~ year, data=RHDand2)
summary(n2and)
```

```
## 
## Call:
## lm(formula = logN ~ year, data = RHDand2)
## 
## Residuals:
##       Min        1Q    Median        3Q       Max 
## -0.086977 -0.007341  0.003848  0.022685  0.044551 
## 
## Coefficients:
##               Estimate Std. Error t value Pr(>|t|)    
## (Intercept) 124.321294  13.413533   9.268 8.92e-05 ***
## year         -0.054685   0.006658  -8.213 0.000176 ***
## ---
## Signif. codes:  0 '***' 0.001 '**' 0.01 '*' 0.05 '.' 0.1 ' ' 1
## 
## Residual standard error: 0.04315 on 6 degrees of freedom
## Multiple R-squared:  0.9183, Adjusted R-squared:  0.9047 
## F-statistic: 67.45 on 1 and 6 DF,  p-value: 0.0001758
```

We found a significant negative linear trend on the log-scale of the
number of yearly hunted rabbits after the GI.2 outbreak (slope = -0.055,
P = 1.8^{-4}).

## Aragon

```
RHDara1<- Aragon[Aragon$year>1987&Aragon$year<1996,]
RHDara1<- RHDara1[1:8,]# cutting out the years after the first eight (same number for which we have info on RHDara2)
RHDara2<- Aragon[Aragon$year>2010,]
RHDara1$logN<- log(RHDara1$N)
RHDara2$logN<- log(RHDara2$N)
n1ara<- lm(logN ~ year, data=RHDara1)
summary(n1ara)
```

```
## 
## Call:
## lm(formula = logN ~ year, data = RHDara1)
## 
## Residuals:
##     Min      1Q  Median      3Q     Max 
## -0.7606 -0.3794 -0.1309  0.2732  1.2584 
## 
## Coefficients:
##             Estimate Std. Error t value Pr(>|t|)
## (Intercept) 207.2971   207.5357   0.999    0.356
## year         -0.0990     0.1042  -0.950    0.379
## 
## Residual standard error: 0.6754 on 6 degrees of freedom
## Multiple R-squared:  0.1307, Adjusted R-squared:  -0.01413 
## F-statistic: 0.9024 on 1 and 6 DF,  p-value: 0.3788
```

We found a non-significant linear trend on the log-scale of the
number of yearly hunted rabbits after the GI.1 outbreak (slope = -0.099,
P = 0.37881).

```
n2ara<- lm(logN ~ year, data=RHDara2)
summary(n2ara)
```

```
## 
## Call:
## lm(formula = logN ~ year, data = RHDara2)
## 
## Residuals:
##       Min        1Q    Median        3Q       Max 
## -0.068401 -0.059385 -0.002427  0.012909  0.162004 
## 
## Coefficients:
##               Estimate Std. Error t value Pr(>|t|)    
## (Intercept) -242.32746   24.92262  -9.723 6.80e-05 ***
## year           0.12678    0.01237  10.247 5.04e-05 ***
## ---
## Signif. codes:  0 '***' 0.001 '**' 0.01 '*' 0.05 '.' 0.1 ' ' 1
## 
## Residual standard error: 0.08018 on 6 degrees of freedom
## Multiple R-squared:  0.9459, Adjusted R-squared:  0.9369 
## F-statistic:   105 on 1 and 6 DF,  p-value: 5.038e-05
```

We found a significant positive linear trend on the log-scale of the
number of yearly hunted rabbits after the GI.2 outbreak (slope = 0.127,
P = 5^{-5}).

## Balearic Islands

```
RHDbal1<- Baleares[Baleares$year>1987&Baleares$year<1996,]
RHDbal1<- RHDbal1[1:8,]# cutting out the years after the first eight (same number for which we have info on RHDbal2)
RHDbal2<- Baleares[Baleares$year>2010,]
RHDbal1$logN<- log(RHDbal1$N)
RHDbal2$logN<- log(RHDbal2$N)
n1bal<- lm(logN ~ year, data=RHDbal1)
summary(n1bal)
```

```
## 
## Call:
## lm(formula = logN ~ year, data = RHDbal1)
## 
## Residuals:
##     Min      1Q  Median      3Q     Max 
## -0.3919 -0.1385 -0.0665  0.1401  0.4412 
## 
## Coefficients:
##             Estimate Std. Error t value Pr(>|t|)   
## (Intercept) 495.3966    89.6108   5.528  0.00148 **
## year         -0.2424     0.0450  -5.387  0.00168 **
## ---
## Signif. codes:  0 '***' 0.001 '**' 0.01 '*' 0.05 '.' 0.1 ' ' 1
## 
## Residual standard error: 0.2916 on 6 degrees of freedom
## Multiple R-squared:  0.8287, Adjusted R-squared:  0.8001 
## F-statistic: 29.02 on 1 and 6 DF,  p-value: 0.001685
```

We found a significant negative linear trend on the log-scale of the
number of yearly hunted rabbits after the GI.1 outbreak (slope = -0.242,
P = 0.00168).

```
n2bal<- lm(logN ~ year, data=RHDbal2)
summary(n2bal)
```

```
## 
## Call:
## lm(formula = logN ~ year, data = RHDbal2)
## 
## Residuals:
##      Min       1Q   Median       3Q      Max 
## -0.45595 -0.05685  0.04203  0.14448  0.24238 
## 
## Coefficients:
##             Estimate Std. Error t value Pr(>|t|)
## (Intercept) 67.10632   75.68969   0.887    0.409
## year        -0.02768    0.03757  -0.737    0.489
## 
## Residual standard error: 0.2435 on 6 degrees of freedom
## Multiple R-squared:  0.08296,    Adjusted R-squared:  -0.06988 
## F-statistic: 0.5428 on 1 and 6 DF,  p-value: 0.4891
```

We found a non-significant linear trend on the log-scale of the
number of yearly hunted rabbits after the GI.2 outbreak (slope = -0.028,
P = 0.48906).

## Basque Country

```
RHDpai1<- PaisVasco[PaisVasco$year>1987&PaisVasco$year<1996,]
RHDpai1<- RHDpai1[1:8,]# cutting out the years after the first eight (same number for which we have info on RHDpai2)
RHDpai2<- PaisVasco[PaisVasco$year>2010,]
RHDpai1$logN<- log(RHDpai1$N)
RHDpai2$logN<- log(RHDpai2$N)
n1pai<- lm(logN ~ year, data=RHDpai1)
summary(n1pai)
```

```
## 
## Call:
## lm(formula = logN ~ year, data = RHDpai1)
## 
## Residuals:
##      Min       1Q   Median       3Q      Max 
## -0.29173 -0.14836  0.04382  0.11780  0.23453 
## 
## Coefficients:
##              Estimate Std. Error t value Pr(>|t|)   
## (Intercept) 268.68051   65.18126   4.122  0.00620 **
## year         -0.13074    0.03273  -3.995  0.00716 **
## ---
## Signif. codes:  0 '***' 0.001 '**' 0.01 '*' 0.05 '.' 0.1 ' ' 1
## 
## Residual standard error: 0.2121 on 6 degrees of freedom
## Multiple R-squared:  0.7267, Adjusted R-squared:  0.6812 
## F-statistic: 15.96 on 1 and 6 DF,  p-value: 0.007163
```

We found a significant negative linear trend on the log-scale of the
number of yearly hunted rabbits after the GI.1 outbreak (slope = -0.131,
P = 0.00716).

```
n2pai<- lm(logN ~ year, data=RHDpai2)
summary(n2pai)
```

```
## 
## Call:
## lm(formula = logN ~ year, data = RHDpai2)
## 
## Residuals:
##     Min      1Q  Median      3Q     Max 
## -1.3298 -1.0132 -0.1895  0.8177  1.8273 
## 
## Coefficients:
##               Estimate Std. Error t value Pr(>|t|)  
## (Intercept) -1448.5738   411.2302  -3.523   0.0125 *
## year            0.7226     0.2041   3.540   0.0122 *
## ---
## Signif. codes:  0 '***' 0.001 '**' 0.01 '*' 0.05 '.' 0.1 ' ' 1
## 
## Residual standard error: 1.323 on 6 degrees of freedom
## Multiple R-squared:  0.6762, Adjusted R-squared:  0.6222 
## F-statistic: 12.53 on 1 and 6 DF,  p-value: 0.01222
```

We found a significant positive linear trend on the log-scale of the
number of yearly hunted rabbits after the GI.2 outbreak (slope = 0.723,
P = 0.01222).

## Castille La Mancha

```
RHDclm1<- CastillalaMancha[CastillalaMancha$year>1987&CastillalaMancha$year<1996,]
RHDclm1<- RHDclm1[1:8,]# cutting out the years after the first eight (same number for which we have info on RHDclm2)
RHDclm2<- CastillalaMancha[CastillalaMancha$year>2010,]
RHDclm1$logN<- log(RHDclm1$N)
RHDclm2$logN<- log(RHDclm2$N)
n1clm<- lm(logN ~ year, data=RHDclm1)
summary(n1clm)
```

```
## 
## Call:
## lm(formula = logN ~ year, data = RHDclm1)
## 
## Residuals:
##      Min       1Q   Median       3Q      Max 
## -0.26644 -0.13445 -0.01862  0.11230  0.35875 
## 
## Coefficients:
##              Estimate Std. Error t value Pr(>|t|)  
## (Intercept) 195.20806   66.02512   2.957   0.0254 *
## year         -0.09119    0.03315  -2.751   0.0333 *
## ---
## Signif. codes:  0 '***' 0.001 '**' 0.01 '*' 0.05 '.' 0.1 ' ' 1
## 
## Residual standard error: 0.2149 on 6 degrees of freedom
## Multiple R-squared:  0.5577, Adjusted R-squared:  0.484 
## F-statistic: 7.566 on 1 and 6 DF,  p-value: 0.03326
```

We found a significant negative linear trend on the log-scale of the
number of yearly hunted rabbits after the GI.1 outbreak (slope = -0.091,
P = 0.03326).

```
n2clm<- lm(logN ~ year, data=RHDclm2)
summary(n2clm)
```

```
## 
## Call:
## lm(formula = logN ~ year, data = RHDclm2)
## 
## Residuals:
##       Min        1Q    Median        3Q       Max 
## -0.199456 -0.056676  0.000915  0.055791  0.182456 
## 
## Coefficients:
##              Estimate Std. Error t value Pr(>|t|)
## (Intercept) 22.039823  39.078138   0.564    0.593
## year        -0.003727   0.019398  -0.192    0.854
## 
## Residual standard error: 0.1257 on 6 degrees of freedom
## Multiple R-squared:  0.006115,   Adjusted R-squared:  -0.1595 
## F-statistic: 0.03692 on 1 and 6 DF,  p-value: 0.854
```

We found a significant positive linear trend on the log-scale of the
number of yearly hunted rabbits after the GI.2 outbreak (slope = -0.004,
P = 0.85397).

## Castille and Leon

```
RHDcyl1<- CastillayLeon[CastillayLeon$year>1987&CastillayLeon$year<1996,]
RHDcyl1<- RHDcyl1[1:8,]# cutting out the years after the first eight (same number for which we have info on RHDcyl2)
RHDcyl2<- CastillayLeon[CastillayLeon$year>2010,]
RHDcyl1$logN<- log(RHDcyl1$N)
RHDcyl2$logN<- log(RHDcyl2$N)
n1cyl<- lm(logN ~ year, data=RHDcyl1)
summary(n1cyl)
```

```
## 
## Call:
## lm(formula = logN ~ year, data = RHDcyl1)
## 
## Residuals:
##     Min      1Q  Median      3Q     Max 
## -0.3489 -0.2531 -0.1271  0.1845  0.6310 
## 
## Coefficients:
##             Estimate Std. Error t value Pr(>|t|)   
## (Intercept) 575.8427   114.1221   5.046  0.00234 **
## year         -0.2832     0.0573  -4.943  0.00260 **
## ---
## Signif. codes:  0 '***' 0.001 '**' 0.01 '*' 0.05 '.' 0.1 ' ' 1
## 
## Residual standard error: 0.3714 on 6 degrees of freedom
## Multiple R-squared:  0.8028, Adjusted R-squared:   0.77 
## F-statistic: 24.43 on 1 and 6 DF,  p-value: 0.002597
```

We found a significant negative linear trend on the log-scale of the
number of yearly hunted rabbits after the GI.1 outbreak (slope = -0.283,
P = 0.0026).

```
n2cyl<- lm(logN ~ year, data=RHDcyl2)
summary(n2cyl)
```

```
## 
## Call:
## lm(formula = logN ~ year, data = RHDcyl2)
## 
## Residuals:
##       Min        1Q    Median        3Q       Max 
## -0.256154 -0.056491  0.007184  0.112284  0.167896 
## 
## Coefficients:
##               Estimate Std. Error t value Pr(>|t|)  
## (Intercept) -108.39941   49.45120  -2.192   0.0709 .
## year           0.06003    0.02455   2.446   0.0501 .
## ---
## Signif. codes:  0 '***' 0.001 '**' 0.01 '*' 0.05 '.' 0.1 ' ' 1
## 
## Residual standard error: 0.1591 on 6 degrees of freedom
## Multiple R-squared:  0.4992, Adjusted R-squared:  0.4157 
## F-statistic: 5.981 on 1 and 6 DF,  p-value: 0.05009
```

We found a marginally significant positive linear trend on the
log-scale of the number of yearly hunted rabbits after the GI.2 outbreak
(slope = 0.06, P = 0.05009).

## Catalonia

```
RHDcat1<- Cataluna[Cataluna$year>1987&Cataluna$year<1996,]
RHDcat1<- RHDcat1[1:8,]# cutting out the years after the first eight (same number for which we have info on RHDcat2)
RHDcat2<- Cataluna[Cataluna$year>2010,]
RHDcat1$logN<- log(RHDcat1$N)
RHDcat2$logN<- log(RHDcat2$N)
n1cat<- lm(logN ~ year, data=RHDcat1)
summary(n1cat)
```

```
## 
## Call:
## lm(formula = logN ~ year, data = RHDcat1)
## 
## Residuals:
##       Min        1Q    Median        3Q       Max 
## -0.151111  0.002862  0.027594  0.030554  0.038467 
## 
## Coefficients:
##              Estimate Std. Error t value Pr(>|t|)    
## (Intercept) 128.03924   21.05284   6.082 0.000898 ***
## year         -0.05823    0.01057  -5.508 0.001503 ** 
## ---
## Signif. codes:  0 '***' 0.001 '**' 0.01 '*' 0.05 '.' 0.1 ' ' 1
## 
## Residual standard error: 0.06851 on 6 degrees of freedom
## Multiple R-squared:  0.8349, Adjusted R-squared:  0.8074 
## F-statistic: 30.34 on 1 and 6 DF,  p-value: 0.001503
```

We found a significant negative linear trend on the log-scale of the
number of yearly hunted rabbits after the GI.1 outbreak (slope = -0.058,
P = 0.0015).

```
n2cat<- lm(logN ~ year, data=RHDcat2)
summary(n2cat)
```

```
## 
## Call:
## lm(formula = logN ~ year, data = RHDcat2)
## 
## Residuals:
##       Min        1Q    Median        3Q       Max 
## -0.184985 -0.068587 -0.002701  0.074970  0.149003 
## 
## Coefficients:
##              Estimate Std. Error t value Pr(>|t|)
## (Intercept)  1.058014  38.678873   0.027    0.979
## year         0.005499   0.019200   0.286    0.784
## 
## Residual standard error: 0.1244 on 6 degrees of freedom
## Multiple R-squared:  0.01349,    Adjusted R-squared:  -0.1509 
## F-statistic: 0.08204 on 1 and 6 DF,  p-value: 0.7842
```

We found a non-significant linear trend on the log-scale of the
number of yearly hunted rabbits after the GI.2 outbreak (slope = 0.005,
P = 0.78418).

## Extremadura

```
RHDext1<- Extremadura[Extremadura$year>1987&Extremadura$year<1996,]
RHDext1<- RHDext1[1:8,]# cutting out the years after the first eight (same number for which we have info on RHDext2)
RHDext2<- Extremadura[Extremadura$year>2010,]
RHDext1$logN<- log(RHDext1$N)
RHDext2$logN<- log(RHDext2$N)
n1ext<- lm(logN ~ year, data=RHDext1)
summary(n1ext)
```

```
## 
## Call:
## lm(formula = logN ~ year, data = RHDext1)
## 
## Residuals:
##      Min       1Q   Median       3Q      Max 
## -0.09435 -0.05718 -0.03851  0.02370  0.22717 
## 
## Coefficients:
##              Estimate Std. Error t value Pr(>|t|)  
## (Intercept) -53.89605   34.45306  -1.564   0.1688  
## year          0.03383    0.01730   1.955   0.0983 .
## ---
## Signif. codes:  0 '***' 0.001 '**' 0.01 '*' 0.05 '.' 0.1 ' ' 1
## 
## Residual standard error: 0.1121 on 6 degrees of freedom
## Multiple R-squared:  0.3892, Adjusted R-squared:  0.2874 
## F-statistic: 3.824 on 1 and 6 DF,  p-value: 0.09831
```

We found a non-significant linear trend on the log-scale of the
number of yearly hunted rabbits after the GI.1 outbreak (slope = 0.034,
P = 0.09831).

```
n2ext<- lm(logN ~ year, data=RHDext2)
summary(n2ext)
```

```
## 
## Call:
## lm(formula = logN ~ year, data = RHDext2)
## 
## Residuals:
##       Min        1Q    Median        3Q       Max 
## -0.243691 -0.112524 -0.004564  0.113688  0.279717 
## 
## Coefficients:
##              Estimate Std. Error t value Pr(>|t|)    
## (Intercept) 366.95948   61.05475   6.010 0.000956 ***
## year         -0.17658    0.03031  -5.826 0.001125 ** 
## ---
## Signif. codes:  0 '***' 0.001 '**' 0.01 '*' 0.05 '.' 0.1 ' ' 1
## 
## Residual standard error: 0.1964 on 6 degrees of freedom
## Multiple R-squared:  0.8498, Adjusted R-squared:  0.8248 
## F-statistic: 33.95 on 1 and 6 DF,  p-value: 0.001125
```

We found a significant negative linear trend on the log-scale of the
number of yearly hunted rabbits after the GI.2 outbreak (slope = -0.177,
P = 0.00112).

## Galicia

```
RHDgal1<- Galicia[Galicia$year>1987&Galicia$year<1996,]
RHDgal1<- RHDgal1[1:8,]# cutting out the years after the first eight (same number for which we have info on RHDgal2)
RHDgal2<- Galicia[Galicia$year>2010,]
RHDgal1$logN<- log(RHDgal1$N)
RHDgal2$logN<- log(RHDgal2$N)
n1gal<- lm(logN ~ year, data=RHDgal1)
summary(n1gal)
```

```
## 
## Call:
## lm(formula = logN ~ year, data = RHDgal1)
## 
## Residuals:
##      Min       1Q   Median       3Q      Max 
## -0.14398 -0.06837 -0.05484  0.07470  0.21995 
## 
## Coefficients:
##              Estimate Std. Error t value Pr(>|t|)
## (Intercept) 18.639033  39.975196   0.466    0.657
## year        -0.003274   0.020073  -0.163    0.876
## 
## Residual standard error: 0.1301 on 6 degrees of freedom
## Multiple R-squared:  0.004414,   Adjusted R-squared:  -0.1615 
## F-statistic: 0.0266 on 1 and 6 DF,  p-value: 0.8758
```

We found a non-significant linear trend on the log-scale of the
number of yearly hunted rabbits after the GI.1 outbreak (slope = -0.003,
P = 0.8758).

```
n2gal<- lm(logN ~ year, data=RHDgal2)
summary(n2gal)
```

```
## 
## Call:
## lm(formula = logN ~ year, data = RHDgal2)
## 
## Residuals:
##      Min       1Q   Median       3Q      Max 
## -0.28512 -0.17945 -0.01267  0.07485  0.43648 
## 
## Coefficients:
##              Estimate Std. Error t value Pr(>|t|)
## (Intercept) 137.31955   82.70364   1.660    0.148
## year         -0.06258    0.04105  -1.524    0.178
## 
## Residual standard error: 0.2661 on 6 degrees of freedom
## Multiple R-squared:  0.2792, Adjusted R-squared:  0.1591 
## F-statistic: 2.324 on 1 and 6 DF,  p-value: 0.1782
```

We found a non-significant linear trend on the log-scale of the
number of yearly hunted rabbits after the GI.2 outbreak (slope = -0.063,
P = 0.17824).

## La Rioja

```
RHDlrj1<- LaRioja[LaRioja$year>1987&LaRioja$year<1996,]
RHDlrj1<- RHDlrj1[1:8,]# cutting out the years after the first eight (same number for which we have info on RHDlrj2)
RHDlrj2<- LaRioja[LaRioja$year>2010,]
RHDlrj1$logN<- log(RHDlrj1$N)
RHDlrj2$logN<- log(RHDlrj2$N)
n1lrj<- lm(logN ~ year, data=RHDlrj1)
summary(n1lrj)
```

```
## 
## Call:
## lm(formula = logN ~ year, data = RHDlrj1)
## 
## Residuals:
##       Min        1Q    Median        3Q       Max 
## -0.112929 -0.005660 -0.002662  0.010353  0.086652 
## 
## Coefficients:
##              Estimate Std. Error t value Pr(>|t|)
## (Intercept) 12.079504  18.745784   0.644    0.543
## year        -0.001090   0.009413  -0.116    0.912
## 
## Residual standard error: 0.061 on 6 degrees of freedom
## Multiple R-squared:  0.002231,   Adjusted R-squared:  -0.1641 
## F-statistic: 0.01341 on 1 and 6 DF,  p-value: 0.9116
```

We found a non-significant linear trend on the log-scale of the
number of yearly hunted rabbits after the GI.1 outbreak (slope = -0.001,
P = 0.91157).

```
n2lrj<- lm(logN ~ year, data=RHDlrj2)
summary(n2lrj)
```

```
## 
## Call:
## lm(formula = logN ~ year, data = RHDlrj2)
## 
## Residuals:
##      Min       1Q   Median       3Q      Max 
## -0.23375 -0.22404 -0.05275  0.12896  0.49609 
## 
## Coefficients:
##              Estimate Std. Error t value Pr(>|t|)
## (Intercept) -59.19775   89.52360  -0.661    0.533
## year          0.03500    0.04444   0.788    0.461
## 
## Residual standard error: 0.288 on 6 degrees of freedom
## Multiple R-squared:  0.0937, Adjusted R-squared:  -0.05734 
## F-statistic: 0.6204 on 1 and 6 DF,  p-value: 0.4609
```

We found a non-significant linear trend on the log-scale of the
number of yearly hunted rabbits after the GI.2 outbreak (slope = 0.035,
P = 0.46089).

## Madrid

```
RHDmad1<- CastillayLeon[CastillayLeon$year>1987&CastillayLeon$year<1996,]
RHDmad1<- RHDmad1[1:8,]# cutting out the years after the first eight (same number for which we have info on RHDmad2)
RHDmad2<- CastillayLeon[CastillayLeon$year>2010,]
RHDmad1$logN<- log(RHDmad1$N)
RHDmad2$logN<- log(RHDmad2$N)
n1mad<- lm(logN ~ year, data=RHDmad1)
summary(n1mad)
```

```
## 
## Call:
## lm(formula = logN ~ year, data = RHDmad1)
## 
## Residuals:
##     Min      1Q  Median      3Q     Max 
## -0.3489 -0.2531 -0.1271  0.1845  0.6310 
## 
## Coefficients:
##             Estimate Std. Error t value Pr(>|t|)   
## (Intercept) 575.8427   114.1221   5.046  0.00234 **
## year         -0.2832     0.0573  -4.943  0.00260 **
## ---
## Signif. codes:  0 '***' 0.001 '**' 0.01 '*' 0.05 '.' 0.1 ' ' 1
## 
## Residual standard error: 0.3714 on 6 degrees of freedom
## Multiple R-squared:  0.8028, Adjusted R-squared:   0.77 
## F-statistic: 24.43 on 1 and 6 DF,  p-value: 0.002597
```

We found a significant negative linear trend on the log-scale of the
number of yearly hunted rabbits after the GI.1 outbreak (slope = -0.283,
P = 0.0026).

```
n2mad<- lm(logN ~ year, data=RHDmad2)
summary(n2mad)
```

```
## 
## Call:
## lm(formula = logN ~ year, data = RHDmad2)
## 
## Residuals:
##       Min        1Q    Median        3Q       Max 
## -0.256154 -0.056491  0.007184  0.112284  0.167896 
## 
## Coefficients:
##               Estimate Std. Error t value Pr(>|t|)  
## (Intercept) -108.39941   49.45120  -2.192   0.0709 .
## year           0.06003    0.02455   2.446   0.0501 .
## ---
## Signif. codes:  0 '***' 0.001 '**' 0.01 '*' 0.05 '.' 0.1 ' ' 1
## 
## Residual standard error: 0.1591 on 6 degrees of freedom
## Multiple R-squared:  0.4992, Adjusted R-squared:  0.4157 
## F-statistic: 5.981 on 1 and 6 DF,  p-value: 0.05009
```

We found a marginally significant positive linear trend on the
log-scale of the number of yearly hunted rabbits after the GI.2 outbreak
(slope = 0.06, P = 0.05009).

## Navarra

```
RHDnav1<- Navarra[Navarra$year>1987&Navarra$year<1996,]
RHDnav1<- RHDnav1[1:8,]# cutting out the years after the first eight (same number for which we have info on RHDnav2)
RHDnav2<- Navarra[Navarra$year>2010,]
RHDnav1$logN<- log(RHDnav1$N)
RHDnav2$logN<- log(RHDnav2$N)
n1nav<- lm(logN ~ year, data=RHDnav1)
summary(n1nav)
```

```
## 
## Call:
## lm(formula = logN ~ year, data = RHDnav1)
## 
## Residuals:
##      Min       1Q   Median       3Q      Max 
## -0.44660 -0.14581 -0.02734  0.09113  0.61063 
## 
## Coefficients:
##              Estimate Std. Error t value Pr(>|t|)
## (Intercept) 145.22495  101.68711   1.428    0.203
## year         -0.06770    0.05106  -1.326    0.233
## 
## Residual standard error: 0.3309 on 6 degrees of freedom
## Multiple R-squared:  0.2266, Adjusted R-squared:  0.09769 
## F-statistic: 1.758 on 1 and 6 DF,  p-value: 0.2331
```

We found a non-significant linear trend on the log-scale of the
number of yearly hunted rabbits after the GI.1 outbreak (slope = -0.068,
P = 0.23313).

```
n2nav<- lm(logN ~ year, data=RHDnav2)
summary(n2nav)
```

```
## 
## Call:
## lm(formula = logN ~ year, data = RHDnav2)
## 
## Residuals:
##      Min       1Q   Median       3Q      Max 
## -0.28999 -0.13800  0.06635  0.13630  0.22062 
## 
## Coefficients:
##              Estimate Std. Error t value Pr(>|t|)
## (Intercept) -11.96488   63.84139  -0.187    0.858
## year          0.01181    0.03169   0.373    0.722
## 
## Residual standard error: 0.2054 on 6 degrees of freedom
## Multiple R-squared:  0.02262,    Adjusted R-squared:  -0.1403 
## F-statistic: 0.1388 on 1 and 6 DF,  p-value: 0.7223
```

We found a non-significant linear trend on the log-scale of the
number of yearly hunted rabbits after the GI.2 outbreak (slope = 0.012,
P = 0.72225).

## Valencian Community

```
RHDval1<- C.Valenciana[C.Valenciana$year>1987&C.Valenciana$year<1996,]
RHDval1<- RHDval1[1:8,]# cutting out the years after the first eight (same number for which we have info on RHDval2)
RHDval2<- C.Valenciana[C.Valenciana$year>2010,]
RHDval1$logN<- log(RHDval1$N)
RHDval2$logN<- log(RHDval2$N)
n1val<- lm(logN ~ year, data=RHDval1)
summary(n1val)
```

```
## 
## Call:
## lm(formula = logN ~ year, data = RHDval1)
## 
## Residuals:
##      Min       1Q   Median       3Q      Max 
## -0.08781 -0.06794  0.01583  0.05436  0.08429 
## 
## Coefficients:
##             Estimate Std. Error t value Pr(>|t|)  
## (Intercept) 50.08158   22.56531   2.219   0.0683 .
## year        -0.01894    0.01133  -1.672   0.1456  
## ---
## Signif. codes:  0 '***' 0.001 '**' 0.01 '*' 0.05 '.' 0.1 ' ' 1
## 
## Residual standard error: 0.07343 on 6 degrees of freedom
## Multiple R-squared:  0.3178, Adjusted R-squared:  0.2041 
## F-statistic: 2.795 on 1 and 6 DF,  p-value: 0.1456
```

We found a non-significant linear trend on the log-scale of the
number of yearly hunted rabbits after the GI.1 outbreak (slope = -0.019,
P = 0.14557).

```
n2val<- lm(logN ~ year, data=RHDval2)
summary(n2val)
```

```
## 
## Call:
## lm(formula = logN ~ year, data = RHDval2)
## 
## Residuals:
##       Min        1Q    Median        3Q       Max 
## -0.101425 -0.037851 -0.005208  0.029501  0.140244 
## 
## Coefficients:
##              Estimate Std. Error t value Pr(>|t|)  
## (Intercept) -80.79378   27.32568  -2.957   0.0254 *
## year          0.04649    0.01356   3.427   0.0140 *
## ---
## Signif. codes:  0 '***' 0.001 '**' 0.01 '*' 0.05 '.' 0.1 ' ' 1
## 
## Residual standard error: 0.08791 on 6 degrees of freedom
## Multiple R-squared:  0.6619, Adjusted R-squared:  0.6056 
## F-statistic: 11.75 on 1 and 6 DF,  p-value: 0.01401
```

We found a significant positive linear trend on the log-scale of the
number of yearly hunted rabbits after the GI.2 outbreak (slope = 0.046,
P = 0.01401).
